# The Kil Peptide of Bacteriophage λ Blocks *Escherichia coli* Cytokinesis via ZipA-Dependent Inhibition of FtsZ Assembly

**DOI:** 10.1371/journal.pgen.1004217

**Published:** 2014-03-20

**Authors:** Daniel P. Haeusser, Marina Hoashi, Anna Weaver, Nathan Brown, James Pan, James A. Sawitzke, Lynn C. Thomason, Donald L. Court, William Margolin

**Affiliations:** 1 Department of Microbiology and Molecular Genetics, University of Texas Medical School at Houston, Houston, Texas, United States of America; 2 National Cancer Institute at Frederick, Gene Regulation and Chromosome Biology Laboratory, Frederick, Maryland, United States of America; 3 Frederick National Laboratory for Cancer Research, Leidos Biomedical, Inc., Gene Regulation and Chromosome Biology Laboratory, Frederick, Maryland, United States of America; Indiana University, United States of America

## Abstract

Assembly of the essential, tubulin-like FtsZ protein into a ring-shaped structure at the nascent division site determines the timing and position of cytokinesis in most bacteria and serves as a scaffold for recruitment of the cell division machinery. Here we report that expression of bacteriophage λ *kil*, either from a resident phage or from a plasmid, induces filamentation of *Escherichia coli* cells by rapid inhibition of FtsZ ring formation. Mutant alleles of *ftsZ* resistant to the Kil protein map to the FtsZ polymer subunit interface, stabilize FtsZ ring assembly, and confer increased resistance to endogenous FtsZ inhibitors, consistent with Kil inhibiting FtsZ assembly. Cells with the normally essential cell division gene *zipA* deleted (in a modified background) display normal FtsZ rings after *kil* expression, suggesting that ZipA is required for Kil-mediated inhibition of FtsZ rings *in vivo*. In support of this model, point mutations in the C-terminal FtsZ-interaction domain of ZipA abrogate Kil activity without discernibly altering FtsZ-ZipA interactions. An affinity-tagged-Kil derivative interacts with both FtsZ and ZipA, and inhibits sedimentation of FtsZ filament bundles *in vitro*. Together, these data inspire a model in which Kil interacts with FtsZ and ZipA in the cell to prevent FtsZ assembly into a coherent, division-competent ring structure. Phage growth assays show that *kil^+^* phage lyse ∼30% later than *kil* mutant phage, suggesting that Kil delays lysis, perhaps via its interaction with FtsZ and ZipA.

## Author Summary

Bacterial antibiotic resistance is a serious concern, particularly its role in hospital-acquired infection. Viruses that infect bacteria (bacteriophage) can kill their host, and some prevent the bacterial cell from reproducing during that process. Since their discovery, phage have been considered a potential tool against bacterial infection, but little is known regarding how phage-encoded factors may inhibit bacterial cell division. Understanding the interaction between phage factors and the targeted host systems is therefore a critical research goal. Our report focuses on *E. coli* and λ, a well-studied phage that infects it. λ contains a gene, *kil*, whose expression prevents *E. coli* from dividing, causing cells to grow into long filaments that die. Here we report that Kil protein prevents an essential bacterial protein, FtsZ, from properly assembling into the structure needed for cell division. Our data show that Kil can inhibit FtsZ assembly directly *in vitro*, but that ZipA, another essential cell division protein, enhances its activity on FtsZ *in vivo*. The results of our study elucidate one way that a phage naturally inhibits bacterial reproduction, which could serve as a target for rational antibiotic design.

## Introduction

The replication and lytic functions of bacteriophage λ rapidly diminish *Escherichia coli* viability and lead to ultimate host death by lysis [1]. Decades-old research uncovered a secondary mode of λ-induced host cell death using a defective prophage containing only the immunity region and the *P*_L_ operon. Under derepressed conditions, *E. coli* cells containing this defective prophage filament and eventually die.

**Figure 1:**
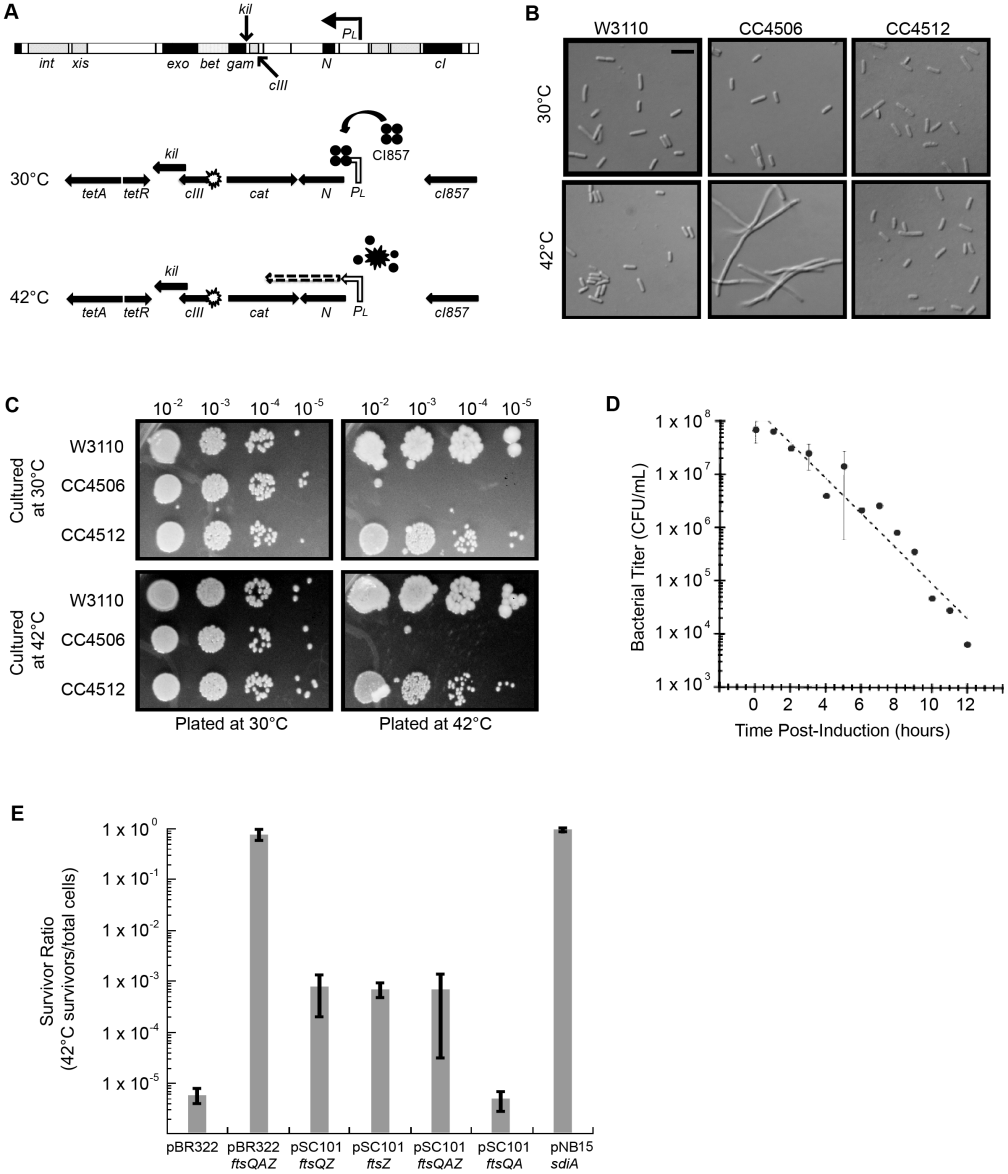
Induction of λ *kil* interferes with *E. coli* cell division, but is suppressed by *ftsZ* overexpression. Strains: W3110 = wild type, CC4506 = *kil^+^*, CC4512 = *kil^−^*. (A) Location of *kil* in the λ genome and the defective prophage used for this study. The λ *kil* gene overlaps *cIII* and *gam* as part of the operon under *P_L_* control (top schematic). *P_L_* expression of *kil* in the defective prophage (CC4506) is under control of the temperature-sensitive *cI857* repressor, allowing repression at 30°C (middle schematic) and expression at 42°C (bottom schematic). (B) Induction of *kil* causes cell filamentation. Differential interference contrast (DIC) microscopy of *E. coli* strains back-diluted from a mid-log 30°C culture to low optical density (OD_600_∼0.025) and grown to late-log in LB at 30°C (top panels) or 42°C (bottom panels). Scale bar = 5 µm. (C) Induction of *kil* leads to a loss in cell viability. Serial spot dilutions of cells grown in LB at 30°C (top panels) or 42°C for 90 minutes (bottom panels) and then plated onto LB agar incubated at 30°C (left panels) or 42°C (right panels) overnight. (D) Prolonged induction of *kil* leads to an irreversible loss of viability. Bacterial titers of CC4506, expressed as colony-forming units (cfu) per mL, after indicated time of *kil* induction. Time points with error bars represent the average and standard deviation between three replicate experiments. (E) Over-expression of the *E. coli* cell division gene *ftsZ* suppresses the loss of cell viability caused by *kil* induction. Plating efficiencies expressed as survivor ratios (cfu/mL surviving at 42°C divided by total cells) for CC4506 with indicated plasmids containing different combinations of the genes in the *ftsQAZ* operon. Error bars represent the standard deviation between three replicate experiments.

The *P*_L_ operon (Figure 1A, top) contains no genes essential for lytic growth except *N*, but consists of accessory genes that may be essential in certain circumstances, such as the *red* recombination genes (*exo*, *bet*, and *gam*) [2]. A series of nested deletions beginning at *attL* and *int*, and removing successive prophage genes toward *P_L_*, initially defined the region of this secondary, lysis-independent killing function [3]. These deletions identified a putative gene located in this region named *kil* (host killing by an induced λ prophage) responsible for host cell filamentation, loss of viability, and ultimate death. However, because *kil* overlaps the *gam* and *cIII* genes [4], some questions remained concerning the exact identity of *kil* and the possible influence of *gam* and *cIII* on host killing [2], [5]. Later experiments further mapped Kil activity to the annotated *kil* open reading frame [6]. Additionally, separate experiments inducing expression of the annotated *kil* open reading frame from a plasmid verified that this region was responsible for causing cell filamentation and a loss of viability [7].

Although assumed to encode a protein, the product of the annotated *kil* gene has not been identified. Likewise, the host cell target of the *kil* gene product (Kil) is unknown. Given the strong cell filamentation and loss-of-viability phenotypes associated with *kil* expression, we reasoned that Kil likely targets a component of the *E. coli* cell division apparatus.

Cytokinesis in most bacteria studied to date involves assembly of the highly conserved prokaryotic tubulin-homolog FtsZ into a ring-shaped structure at the nascent site of divison [8]. FtsZ undergoes GTP-dependent polymerization into single stranded polymers *in vitro*, which in turn are able to bundle together through lateral interactions that are enhanced by certain buffer conditions or bundling agents [9].

In *E. coli*, the assembly of the FtsZ ring at mid-cell prior to division is stabilized and linked to the membrane through the formation of a ‘proto-ring’ that includes the essential transmembrane-anchored ZipA and membrane-associated FtsA proteins [10]. Once the proto-ring is formed, a coterie of both essential and non-essential proteins is recruited in a partially step-wise fashion to form a mature complex termed the divisome. The divisome contains all the components necessary to divide the cell through a combination of constriction, a switch from lateral cell wall growth to septum (crosswall) formation, and cell separation [11].

Cell survival requires proper coordination of division with other cell cycle events, such as growth, DNA replication, and chromosome segregation. This coordination is largely controlled by precisely regulating the timing and position of FtsZ ring formation by altering FtsZ assembly dynamics [12]. In *E. coli*, this regulation is chiefly controlled through the combined activities of the Min system (MinCDE) and the nucleoid-occlusion factor SlmA [8], [13]. SlmA is a protein that binds specific regions of the chromosome and prevents FtsZ from assembling over unsegregated nucleoids [14], [15], [16], [17], [18]. The Min system functions to prevent FtsZ from assembling in DNA-free regions of cell poles through the inhibitory activity of MinC, whose localization is controlled by MinD and MinE [13], [19], [20],[21]. Another well-described inhibitor of FtsZ assembly is SulA, which is activated following DNA damage as part of the SOS response to inhibit cell division until genetic errors are corrected [22], [23], [24].

Whereas numerous additional host factors that regulate cell division have been described in several species [11], [25], little work has been done characterizing potential regulation by phage factors. Rac prophage and bacteriophage Mu each contain a gene also named *kil* [26], [27]. Despite their identical names, however, these *kil* genes and their predicted peptide products have no significant similarity to one another or to λ *kil*. Rac *kil* expression prevents FtsZ ring formation, which blocks cell division resulting in filamentation; no details on the direct target or the mechanism of its activity have been reported [26]. In contrast, expression of the Mu *kil* gene results in spherical *E. coli* cells [27], indicative of inhibiting cell elongation by this temperate member of the *Myoviridae*. The cryptic lambdoid prophage Qin (Kim) and its widespread relatives also contain two factors known to affect cell division [28]: DicB, which acts in place of MinD to bring MinC into position to inhibit FtsZ ring formation [29], [30], and *dicF*, which encodes an antisense RNA that inhibits *ftsZ* translation [31]. Finally, the cryptic e14 phage-like element of *E. coli* contains the *sfiC* gene, which inhibits FtsZ assembly following SOS induction similarly to SulA (SfiA) [32], [33], [34].

Here, we report that λ *kil* expression from a defective prophage, from a plasmid, or during induction of a complete, lytic-competent λ lysogen inhibits cell division due to a block in FtsZ ring formation. We verify that Kil encodes a peptide, and show that it acts independently of well-characterized host systems of FtsZ assembly regulation. We further identify and characterize Kil-resistant *ftsZ* and *zipA* mutant alleles, demonstrate inhibition of FtsZ assembly *in vitro* by an affinity-tagged Kil derivative, and discuss potential models for Kil activity on FtsZ assembly. Finally we address the relevance of *kil* to λ biology, demonstrating that Kil acts during normal lytic growth of λ phage and suggesting that this activity can delay cell lysis.

## Results

### Expression of *kil* interferes with *E. coli* cell division

To investigate the role of *kil* expression on *E. coli* cell division, we first used a bacterial strain (CC4506) harboring a defective λ prophage in which a modified operon is under control of a temperature-sensitive (*ts*) allele (*c*I*857*) of the phage CI repressor. In this *kil^+^* strain, a point mutation destroys the start codon of *cIII*, a *cat* cassette replaces *sieB* to *ea10*, and a *tet* cassette replaces *gam* through *int* (Figure 1A). Consistent with previous findings [6], CC4506 cells formed long, non-dividing filaments after derepression of the *P_L_* operon by thermo-inactivation of the CI857 repressor at 42°C (Figure 1B), and lost viability (Figure 1C). In contrast, an isogenic strain with the start codon of *kil* destroyed by a point mutation (CC4512), continued to divide normally following induction of the prophage and retained viability, similar to the W3110 background strain (Figure 1B & C).

The loss of cell viability, presumably caused by the division blockage elicited by *kil* expression, could be rescued in the short term by returning *E. coli* filaments to low temperature conditions that restored CI857 repressor activity (Figure 1C, bottom left). However, prolonged *kil* expression at 42°C (∼6 hours) prevented restoration of growth even after returning cells to conditions that repress *kil* expression (Figure 1D).

To identify the potential target of *kil* expression, we screened a pBR322 library of *E. coli* chromosomal fragments for multi-copy suppression of *kil*-induced toxicity at 42°C. Consistent with *kil* expression targeting cell division, multi-copy expression of an ∼5.4-kb fragment including the *ftsQAZ* operon completely suppressed toxicity. Similarly, low-copy (pSC101) expression of the *ftsQAZ* operon alone suppressed *kil*-induced toxicity, but less efficiently (Figure 1E). Analysis of the *kil*-expressing strain containing pSC101 with *ftsQZ, ftsQA, or ftsZ* alone confirmed that this suppression derives specifically from *ftsZ* (but not *ftsQA*) over-expression. Further supporting the link between the toxicity of *kil* expression and cell division, an independent multi-copy suppressor screen identified *sdiA* (Figure 1E), whose product increases *ftsQAZ* operon transcription [35].

### Kil inhibits FtsZ ring formation

Filamentation of *E. coli* cells could arise either from a failure to form FtsZ rings at the nascent division site, or from a defect in divisome maturation and septal synthesis after FtsZ ring assembly. Over-expression of *ftsZ* could conceivably help suppress either of these mechanisms to permit Kil-resistance. We therefore determined by immunofluorescence microscopy whether FtsZ rings continued to form in *E. coli* cells following derepression of *kil* under *P_L_* control.

**Figure 2:**
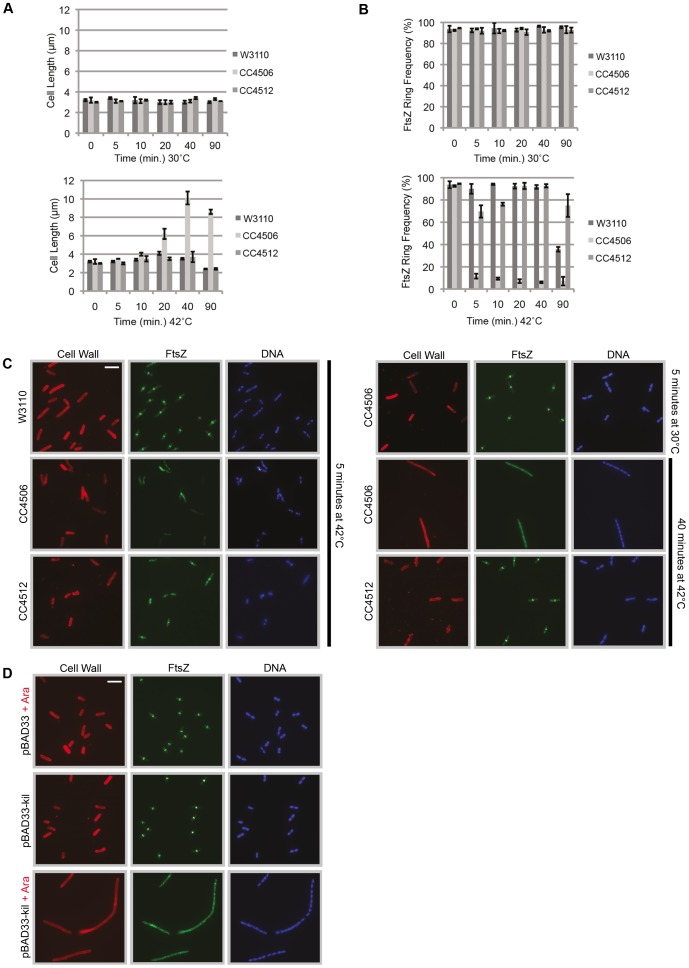
Induction of λ *kil* rapidly blocks FtsZ ring formation. (A) Induction of *kil* causes cell filamentation. Average cell lengths (µm) at various time points (min) following temperature shift to 42°C (bottom panel) or continued incubation (control) at 30°C (top panel), measured from IFM micrographs. Error bars represent standard deviation between average length measurements from three separate images. (B & C) Midcell FtsZ ring localization is rapidly lost upon *kil* induction. (B) FtsZ ring frequency (%) at various time points (min) following temperature shift to 42°C (bottom panel) or continued incubation (control) at 30°C (top panel), counted from IFM micrographs. Error bars represent standard deviation between FtsZ ring frequency counts from three separate images. Cell length and ring frequency measurements at each point were obtained using a minimum number of 100 cells. (C) Representative IFM micrographs of indicated strains at indicated times and temperature from images used for measurements/counts in (A) & (B). Cell wall signal is from rhodamine-conjugated wheat-germ agglutinin, FtsZ signal is from AlexaFluor 488-conjugated, goat α–rabbit recognition of rabbit α-FtsZ, and DNA signal is from 4′,6-diamidino-2-phenylindole (DAPI). Scale bar = 5 µm. (D) Induction of *kil* from pBAD33 in W3110 (lacking λ phage) blocks FtsZ ring formation and leads to cell filamentation. Shown are representative IFM micrographs of W3110 cells containing indicated plasmid under indicated induction conditions. Signals and scale bar are as in (C).

At 30°C, when *P_L_* is repressed, both CC4506 (*kil^+^*) and CC4512 (*kil^−^*) cells were normal-sized on average (3.1±0.1 and 3.1±0.2 µm, respectively) compared to W3110 wild-type control cells (3.1±0.2 µm) (Figure 2A, top). Immunofluorescence microscopy (IFM) with antibody against *E. coli* FtsZ demonstrated that cells of each strain contained a single band of FtsZ signal localized at midcell in the majority (92.4±1.3% to 94.0±1.5%) of the populations, on average (Figure 2B, top).

However, upon a shift to growth at 42°C and the resulting *kil* expression, CC4506 cells continued to grow at a normal rate into nondividing filaments (>10 µm), before reaching stationary phase (Figure 2A, bottom). In contrast, W3110 and CC4512 control strains still displayed normal average cell lengths at 42°C (3.3±0.55 and 3.2±0.48 µm, respectively). Kil induction also led to a rapid (<5 minutes) loss in FtsZ rings observable by IFM, leaving only a few cells with normal FtsZ localization on average (8.3±2.2%). FtsZ localization in W3110 cells was unaffected on average (92.1±1.7%) until 90 minutes post-temperature-shift when cells entered stationary phase and FtsZ ring frequency decreased sharply (Figure 2B, bottom).

Notably, although CC4512 (*kil^−^*) cells displayed normal cell lengths during growth at 42°C (Figure 2A, bottom), this strain background still showed a small, but significant, effect on FtsZ ring formation in the first minutes following temperature shift, with ring frequency dropping to an average of 73.0±4.6%. However, by 20 minutes, these cells had recovered normal FtsZ ring frequencies (92.7±0.07%), unlike the CC4506 *kil^+^* counterpart strain where FtsZ ring frequency remained below 10% (Figure 2B, bottom). This suggests that some other factor in CC4512 has a minor and transient effect on FtsZ assembly upon shift to high temperature.

IFM micrographs corresponding to the data in Figure 2A & B showed normal medial FtsZ localization in both W3110 and CC4512 *kil^−^* strains after five minutes post temperature shift to 42°C (Figure 2C) and all time points observed thereafter (data not shown). CC4506 *kil^+^* cells showed normal FtsZ localization at 30°C, but FtsZ immunostaining became patchy and diffuse after only five minutes of *kil* induction at 42°C. This mislocalization continued throughout growth at 42°C, sometimes forming into broad, unproductive FtsZ foci (possibly helices) along the cell filament (Figure 2C). This pattern, typical upon FtsZ ring formation inhibition *in vivo*, suggests that FtsZ is unable to form a coherent ring-shaped structure in the presence of Kil, thus preventing septum formation and cell division.

Due to the observed *kil*-independent, transient effect on FtsZ ring formation (Figure 2B, bottom), we chose to study the effects of *kil* outside of any phage context by expressing the gene from a plasmid. This enabled us to be certain that any observed effects were caused by Kil alone, and eliminated the need to expose *E. coli* cells to temperature shock. We cloned the *kil* gene downstream of the arabinose-inducible promoter in pBAD33 and transformed the resulting plasmid (pDH104) into W3110. W3110 pDH104 cells grown in non-inducing conditions divided normally, comparable to the W3110 background containing empty vector, indicating that uninduced levels of Kil are low. Consistent with the prophage results, expression of *kil* from the plasmid proved sufficient to induce cell filamentation through a rapid loss of FtsZ ring formation (Figure 2D). Similar results were also obtained by cloning *kil* under P*_lac_* (IPTG-inducible) control in pRR48 (data not shown).

### Kil does not alter FtsZ levels or act through well-characterized endogenous systems of FtsZ assembly inhibition

**Figure 3:**
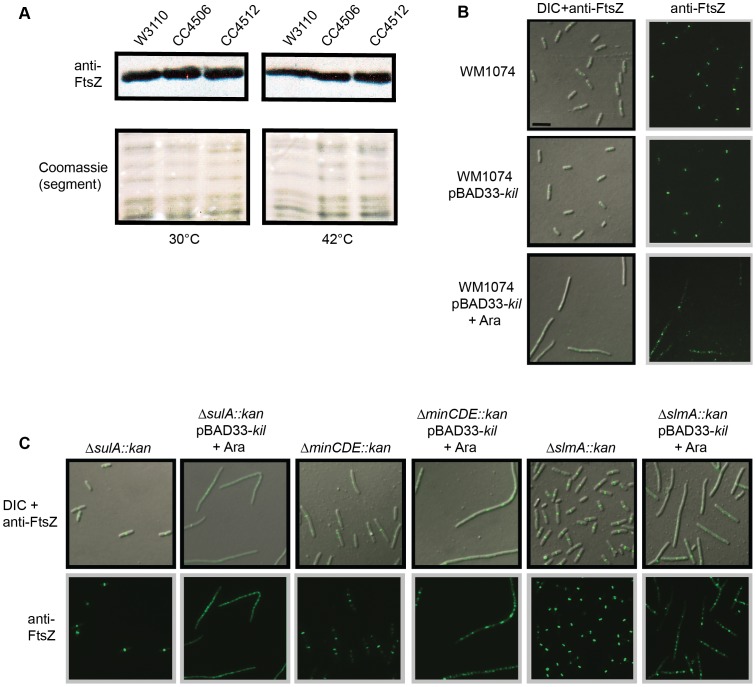
Kil does not alter FtsZ levels or act through SulA, the Min system, or SlmA. (A) FtsZ levels are unchanged by *kil* expression. Shown is an immunoblot against FtsZ (upper panels) from whole cell extracts of indicated strains harvested after 40 minutes of growth at 30°C (left panels) or 42°C (right panels). Relative levels of total protein are shown as a segment of Coomassie stained lanes (bottom panels). (B) Expression of *kil* in an MG1655-derived WM1074 background also blocks FtsZ ring formation and causes cell filamentation. DIC (left panels) and α-FtsZ IFM (left & right panels) micrographs are shown from mid-log cultures (40 minute induction) of indicated strains. (C) Kil-dependent filamentation remains in cells lacking SulA, the Min system, or SlmA (WM1074 background). DIC (top panels) and α-FtsZ IFM (top & bottom panels) micrographs from fixed mid-log cultures (40 min induction) of indicated strains are shown. Scale for micrographs in (B)–(C) is indicated by 5 µm scale bar in (B).

Proper FtsZ assembly and subsequent cell division in *E. coli* is dependent on levels of FtsZ relative to other division components [36]. Though alterations in FtsZ levels are not a normal part of division regulation in *E. coli* [37], conditions that artificially elevate or reduce available FtsZ result in FtsZ mislocalization and cell filamentation [38]. A trivial explanation for the loss of FtsZ ring formation upon the induction of λ *kil* is that its product, the presumed Kil peptide (see below), somehow alters FtsZ protein levels. A known example of this occurs with the cryptic prophage-derived *dicF*, which encodes a small RNA that blocks *ftsZ* mRNA translation by an antisense mechanism [31]. However, quantitative immunoblotting of FtsZ in CC4506 cells induced for *kil* expression showed levels of FtsZ approximately equivalent to those in uninduced CC4506 counterparts or in W3110 and CC4512 controls (Figure 3A). Furthermore, induction of *kil* from pBAD33 or pRR48 in W3110 similarly had no effect on FtsZ compared to levels seen in uninduced cells or cells with empty plasmid (data not shown). These results suggest that the *kil* product does not act by altering FtsZ levels.

Another possibility is that Kil acts indirectly through an endogenous system of FtsZ assembly inhibition, such as the SOS-response activated SulA [22], [23], [39], [40], MinC [41], [42], [43], [44], or the nucleoid-occlusion factor SlmA [14], [15], [16], [17]. This was important to rule out because another phage-derived *dic* gene, *dicB*, encodes a small protein that inhibits FtsZ ring assembly by mimicking MinD to recruit MinC to midcell [29], [30].

To investigate a possible contribution of the SOS-inducible protein, SulA, in Kil activity, we examined the effects of *kil* induction from pBAD33 in a WM1074 *ΔsulA::kan* background. WM1074 is a wild-type derivative of MG1655, whereas the characterizations of Kil presented above utilized the K12 derivative W3110. Expression of *kil* from pBAD33 in WM1074 caused filamentation and inhibition of FtsZ ring formation (Figure 3B) comparable to that seen in W3110 (Figure 2D). Addition of 0.2% arabinose to *sulA^−^* cells resulted in cell filamentation (Figure 3C) indistinguishable from that seen for the *sulA^+^* background, verifying that Kil does not act through SulA and that it confers a similar phenotype in a different strain background.

As with the *ΔsulA::kan* background, induction of *kil* from pBAD33 in *ΔminCDE::kan* or *ΔslmA::kan* backgrounds also increased cell filamentation (Figure 3C), arguing that neither MinC nor SlmA are involved in Kil activity. As shown previously, *minCDE* mutant cells already have division defects due to the presence of extra FtsZ rings, and are elongated. Upon *kil* induction all FtsZ rings disappear into irregular patchy localization, and the already-long *minCDE* mutant cells grow into even longer filaments. Together these data demonstrate that unlike *dicF* or DicB, the *kil* product does not act through any of the well-characterized endogenous host systems that regulate FtsZ assembly.

### Isolation and characterization of *ftsZ* alleles resistant to *kil* expression

**Figure 4:**
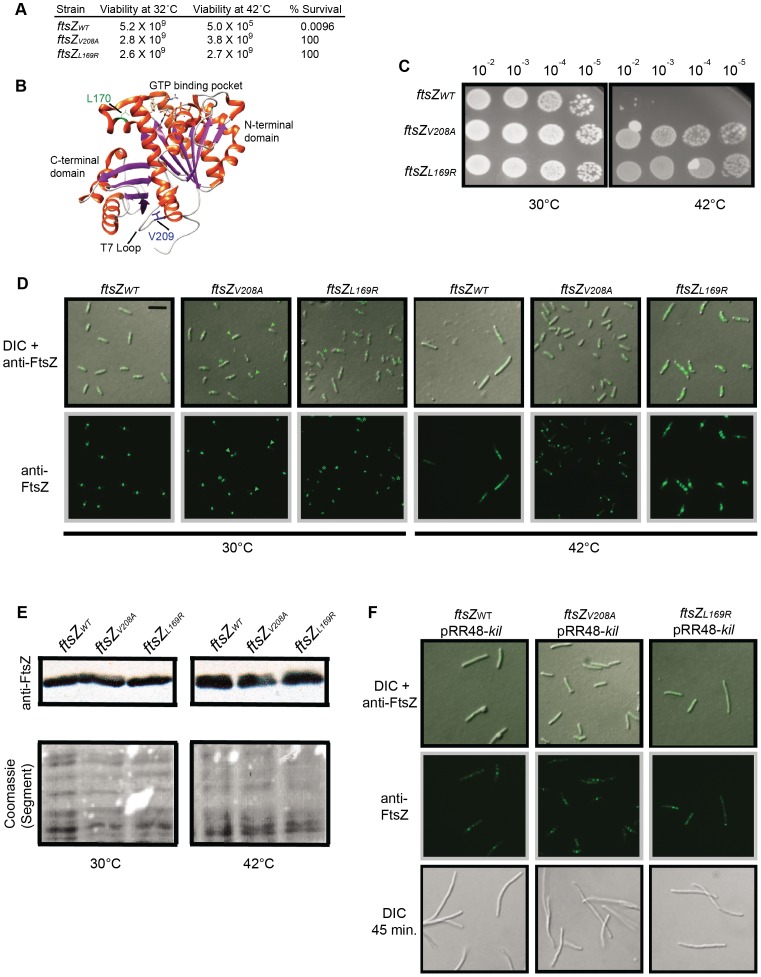
Characterization of Kil-resistant *ftsZ* alleles. (A) The *ftsZ_V208A_* and *ftsZ_L169R_* mutants are Kil-resistant alleles. Shown are cfu at 32°C and 42°C and resulting survivor frequencies (cfu/mL at 42°C divided by cfu/mL at 32°C) for *kil*-expressing strains with *ftsZ_WT_*, *ftsZ_V208A_*, or *ftsZ_L169R_*. (B) Location of L170 and V209 in the crystal structure of *Pseudomonas aeruginosa* FtsZ [92], corresponding to L169 and V208 of *E. coli* FtsZ, generated by Chimera (http://www.cgl.ucsf.edu/chimera/). α-helices are red and β-sheets are purple. The L170 sidechain, adjacent to the GTP-binding pocket, is indicated in green and the V209 sidechain, within the T7 loop, is indicated in blue. (C) Placement of isolated *ftsZ* alleles in a fresh background confers resistance to *kil* expression. Spot dilutions of indicated *ftsZ* alleles in a CC4506 background strain under conditions where *kil* is not expressed (30°C, left) and expressed (42°C, right). (D) Expression of *kil* in strains harboring *ftsZ_V208A_* or *ftsZ_L169R_* does not result in cell filamentation and permits FtsZ ring formation, although mutant FtsZ ring localization is not completely normal upon *kil* expression at 42°C. Representative DIC (top) or α-FtsZ (top & bottom) micrographs of fixed cells are shown with indicated *ftsZ* alleles in the CC4506 background at 30°C (left) and after 40 minutes of *kil* expression at 42°C (right). α-FtsZ signal is as described in Figure 2. Scale bar = 5 µm. For *ftsz_V208A_* cells, arrowheads mark rings or puncta at cell poles and asterisks mark ring doublets. For ftsZ_L169R_ cells, asterisks mark aberrant FtsZ structures. (E) FtsZ_V208A_ and FtsZ_L169R_ are synthesized at comparable levels to FtsZ_WT_. Shown are immunoblots against FtsZ (upper panels) from whole cell extracts of indicated strains harvested after 40 minutes of growth at 30°C (left panels) or 42°C (right panels). Relative levels of total protein are shown as a cropped segment of Coomassie-stained lanes (bottom panels). (F) The amount of Kil produced from a plasmid overcomes the resistance conferred by *ftsZ_V208A_* or *ftsZ_L169R_*. Shown are representative DIC (top and bottom) or α-FtsZ (top & middle) IFM of fixed cells with indicated *ftsZ* alleles in the CC4506 background containing pRR48-*kil* at 30°C, with 1 mM IPTG to induce *kil* from the plasmid for 15 (top and middle) or 45 minutes (bottom). Signal and scale are as in (D).

The preceding results suggest that Kil inhibits FtsZ ring formation, potentially through direct interaction with FtsZ. To further investigate this interaction, we generated *ftsZ* mutant alleles by recombineering with randomly mutagenized *ftsZ* PCR fragments, selecting for those that allowed survival at 42°C in a thermo-inducible *kil^+^* and thermo-sensitive *ftsZ (ftsZ84)* strain (see Materials and Methods). This strategy identified two mutant alleles of *ftsZ* with increased resistance to *kil* expression, *ftsZ_V208A_* and *ftsZ_L169R_* (Figure 4A). According to a recently reported *Mycobacterium tuberculosis* FtsZ crystal structure [45], both of these residues are located in the subunit interface of FtsZ protofilaments. Specifically, FtsZ_V208_ lies in the conserved T7-loop region, which during polymerization inserts into the nucleotide-binding region near the FtsZ_L169_ residue in the N-terminal domain of an adjacent subunit (Figure 4B).

To verify that these alleles confer Kil resistance, we replaced the wild-type *ftsZ* gene of a fresh *kil^+^* strain with each mutant allele. Spot dilutions of the resulting strains verified that the *ftsZ_V208A_* or *ftsZ_L169R_* alleles conferred resistance to Kil compared to the wild-type *ftsZ* allele control, although the appearance of the spots suggests that the strains acquired suppressors fairly readily at 42°C (Figure 4C).

IFM on the *kil^+^* strains harboring *ftsZ_V208A_* or *ftsZ_L169R_* in place of the native wild type allele showed normal-sized cells with FtsZ rings present at 30°C when *kil* expression is repressed (Figure 4D). However, in addition to normal medial ring localization, FtsZ_V208A_ appeared to also form frequent rings or puncta at cell poles (marked by arrowheads) and occasional ring doublets (marked by an asterisk). Similarly, FtsZ_L169R_ sometimes formed aberrant FtsZ structures (marked by asterisks), along the long axis of the cell. These observations suggest that although these FtsZ mutants are competent for assembly and cell division, they seem to be overly stable and insensitive to endogenous regulators of FtsZ assembly.

Unlike in the wild-type *ftsZ* allele background, short-term exposure to *kil* expression at 42°C did not cause cell filamentation in *ftsZ_V208A_* or *ftsZ_L169R_* backgrounds (Figure 4D), consistent with their partial resistance to Kil as seen in spot dilutions (Figure 4C). The apparent insensitivity of the mutant FtsZ_V208A_ and FtsZ_L169R_ proteins to endogenous regulation seen at 30°C is even more obvious at 42°C upon *P_L_* operon derepression. For example, FtsZ_V208A_ localizes predominantly to one cell pole at higher temperature (only ∼14% midcell localization after 40 min of *kil* induction). FtsZ_L169R_, in contrast, forms multiple rings throughout the length of cells at higher temperatures, likely contributing to their slightly longer cell lengths. These cells also contain apparent inclusions visible by DIC microscopy (Figure 4D), which may be from high levels of non-interactive Kil. Importantly, the inclusion bodies and mutant FtsZ localization abnormalities did not arise from inappropriate FtsZ levels in the cell, as both mutant FtsZs were present at levels comparable to the wild-type control (Figure 4E).

The above spot dilution and IFM data demonstrate that the isolated *ftsZ_V208A_* and *ftsZ_L169R_* mutant alleles confer partial resistance to *kil* as expressed from *P_L_* upon de-repression at 42°C, permitting FtsZ assembly, albeit aberrant, and preventing cell filamentation. To further address the Kil-resistance of these mutant *ftsZ* alleles and avoid the mutant FtsZ mislocalization seen at higher temperatures, the above strains were transformed with pRR48-*kil* and investigated by expressing *kil* by IPTG induction at 30°C.

In contrast to the resistance of the *ftsZ* mutant alleles to *kil* expressed at 42°C from *P_L_*, they were unable to confer resistance to short-term induction of *kil* from plasmid pRR48 (1 mM IPTG). Rings formed by FtsZ_WT_, FtsZ_V208A_, or FtsZ_L169R_ were all rapidly lost upon pRR48-*kil* induction (Figure 4F, top and center panels), leading to cell filamentation (Figure 4F, bottom panels). It is possible that the levels of Kil obtained from plasmid expression were higher than those obtained from its native *P_L_* context, but we noticed that longer inductions of *kil* from *P_L_* also ultimately overcame the initial resistance of FtsZ_V208A_ or FtsZ_L169R_ (data not shown). These results suggest that although these *ftsZ* alleles confer partial resistance to Kil from their abnormal assembly characteristics, Kil is still able to overcome these mutant FtsZ proteins under some conditions.

### FtsZ alleles resistant to inhibition by Kil are also resistant to inhibition by MinC or SulA

The abnormal localization of FtsZ_V208_ and FtsZ_L169R_, such as to cell poles, suggested that these mutants may have increased filament stability and a certain general resistance to endogenous FtsZ assembly inhibitors, not just the action of λ Kil. Additionally, WM1074 cells transduced from *ftsZ_WT_* to *ftsZ_V208A_* or *ftsZ_L169R_* had a minicell-producing phenotype (data not shown), further implicating resistance to FtsZ assembly inhibition by MinC.

**Figure 5:**
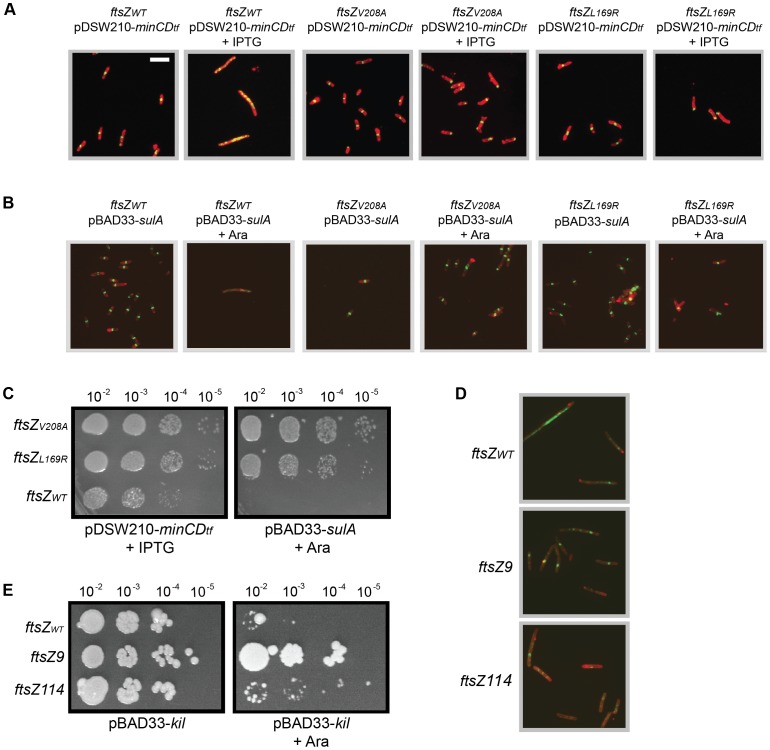
General resistance of isolated ftsZ alleles to assembly inhibition. (A) Cells with *ftsZ_V208A_* or *ftsZ_L169R_* form FtsZ rings and do not filament despite overexpression of *minCD*. Representative IFM micrographs are shown of cell wall (red) and α-FtsZ (green) signal from fixed cells of the CC4506 background strain with pDSW210-*his*-*minCD* (*minC-minD* gene fusion) and the indicated *ftsZ* alleles grown at 30°C (no *kil* induction) harvested after 40 minutes of *minCD* induction (+IPTG) or non-inducing conditions. Cell wall and α-FtsZ signal are shown as in Figure 2. Scale bar = 5 µm. (B) Cells with *ftsZ_V208A_* or *ftsZ_L169R_* form FtsZ rings and do not filament despite *sulA* overexpression. Shown are representative IFM micrographs of cell wall (red) or α-FtsZ (green) signal from fixed cells of the CC4506 background strain with pBAD33-*sulA* and the indicated *ftsZ* alleles grown at 30°C (no *kil* induction) harvested after 40 minutes of *sulA* induction (+Ara) or non-inducing conditions. Signal and scale are as in (A). (C) The *ftsZ* mutations confer resistance to *minCD*- or *sulA*-induced lethality. Spot dilutions are shown of the CC4506 background strain with pDSW210-*his*-*minCD* or pBAD33-*sulA* and the indicated *ftsZ* alleles plated at 30°C (no *kil* expression) on LB agar with 1 mM IPTG (+IPTG) or 0.2% arabinose (+Ara). (D) Cells with the characterized *ftsZ9* and *ftsZ114* alleles partially suppress inhibition of FtsZ ring formation by Kil. Shown are representative IFM micrographs of cell wall (red) or α-FtsZ (green) signal from fixed cells containing the indicated *ftsZ* allele and pBAD33-*kil* grown after 40 minutes of *kil* induction (+Ara). Signal and scale as in (A). (E) Cells with the *ftsZ9* allele completely suppress, and those with the *ftsZ114* allele partially suppress the loss of viability upon *kil* expression. Shown are spot dilutions of cells containing the indicated *ftsZ* allele and pBAD33-*kil* plated on LB agar with (right) or without (left) 0.2% arabinose at the indicated dilutions.

To determine whether the *ftsZ_V208A_* and *ftsZ_L169R_* alleles make the strains generally resistant to FtsZ assembly inhibitors, we monitored resistance to over-expression of *sulA* or to a *his*-*minCD* translational fusion (*minCD*). We transformed the *kil^+^* (under chromosomal *P_L_* control) strains harboring *ftsZ_V208A_* or *ftsZ_L169R_* with either pDSW210-*his*-*minCD* or pBAD33-*sulA* and grew them at 30°C, where *kil* is not expressed whether or not the plasmids are induced. Induction of either *minCD* or *sulA* caused FtsZ_WT_ cell filamentation and a loss of FtsZ ring formation after 40 min. However, in the presence of FtsZ_V208_ or FtsZ_L169R_, high levels of SulA or MinCD had no apparent effect on cell length in the same time frame (Figure 5A & B). Consistent with prior results, FtsZ_V208_ and FtsZ_L169R_ localized normally at 30°C in non-inducing conditions for either plasmid. Similar to observations for the FtsZ mutants upon *kil* expression at 42°C (Figure 4C), induction of *sulA*, and particularly *minCD*, at 30°C caused both FtsZ_V208_ and FtsZ_L169R_ to mislocalize, often at cell poles (Figure 5A & B). This suggests that, in contrast to FtsZ_WT_, both FtsZ mutants are able to assemble in the presence of high SulA or MinCD levels, but that this assembly is abnormal and persists at new cell poles following division, similar to what was seen for these FtsZ mutant proteins in the presence of Kil.

The continued growth and division of *ftsZ_V208A_* or *ftsZ_L169R_* mutant cells upon short-term induction of either *minCD* or *sulA* suggests that these cells remain more viable than wild-type cells, despite many mislocalized FtsZ rings. Spot dilutions of *ftsZ_WT_*, *ftsZ_V208A_*, or *ftsZ_L169R_* cells carrying pDSW210-*his*-*minCD* or pBAD33-*sulA* onto plates with relevant inducers at 30°C (to ensure no *kil* expression) verified that both *ftsZ_V208A_* and *ftsZ_L169R_* increased cell viability upon *sulA* over-expression (Figure 5C). Although *minCD* over-expression blocked wild-type FtsZ ring formation and led to cell filamentation (Figure 5A), these cells remained partially viable in spot dilutions upon full induction with IPTG (1 mM). However, *ftsZ_V208A_* and *ftsZ_L169R_* conferred an approximately 10-fold improvement in that viability (Figure 5C). As expected, cell growth was normal in the absence of inducer (data not shown). As with *kil* resistance (Figure 4D), the *ftsZ_V208A_* allele showed somewhat stronger resistance to *minCD* and *sulA* over-expression compared to the *ftsZ_L169R_* allele (Figure 5C).

Previously characterized *ftsZ* mutant alleles, *ftsZ9* (*ftsZ_18_*_Ω*V-G*_ – a two residue insertion) and *ftsZ114 (ftsZ_F268C_)*, show general resistance to both MinC and SulA [46], [47], [48]. We therefore tested whether these alleles were also resistant to λ *kil* expression. For these experiments we utilized the original PB143 *ftsZ^−^* background strains that contain *ftsZ_WT_*, *ftsZ9*, or *ftsZ114* on a low-copy number plasmid under constitutive expression (pBEF0, pBEF9, and pBEF114) and transformed each with pBAD33-*kil*. FtsZ_WT_ and FtsZ114, which displays ∼50% GTPase activity of the wild-type FtsZ, were unable to resist inhibition by Kil and failed to form detectable FtsZ rings in the presence of Kil. However, FtsZ9, which is nearly devoid of GTPase activity (10% of normal) more strongly resisted Kil inhibition and was able to form some detectable FtsZ rings (Figure 5D). This result in the presence of Kil is comparable to the partial, weak resistance of FtsZ114 (and the relatively strong resistance of FtsZ9) to inhibition by SulA or MinC [46], [47], [48]. The ability of these strains to form colonies with or without *kil* induction correlates well with their Kil resistance by IFM (Figure 5E).

### Kil activity requires ZipA *in vivo*

**Figure 6:**
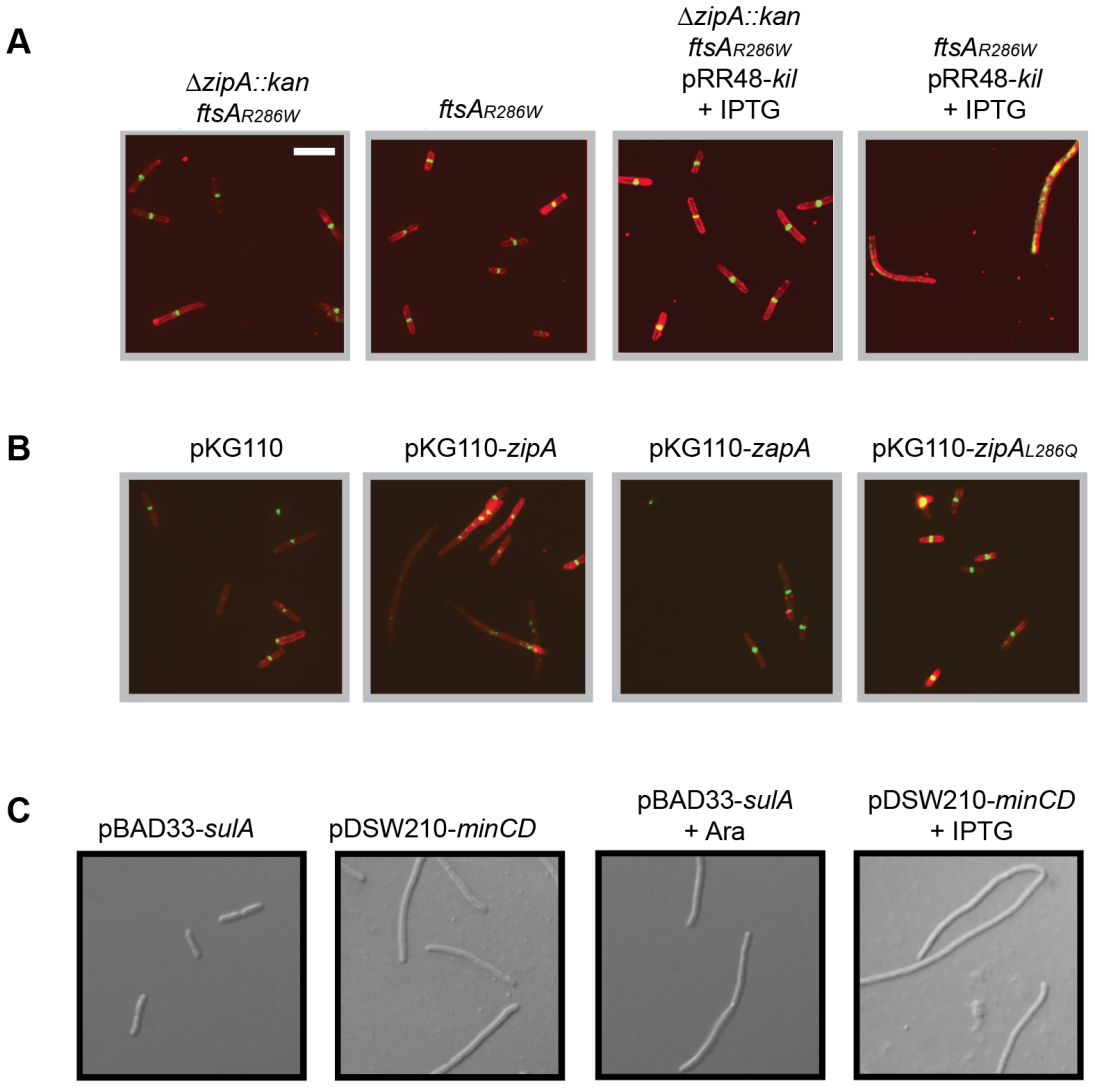
Kil activity *in vivo* requires ZipA. (A) Cells harboring *ftsA_R286W_* to allow bypass of *zipA* (Δ*zipA::aph*) are resistant to *kil* expression, but are not resistant when containing *ftsA_R286W_* alone. Shown are representative IFM micrographs of cell wall (red) or α-FtsZ (green) signal from fixed cells of the indicated genotypes in a WM1074 pRR48-*kil* background with (+IPTG) or without *kil* induction for 40 minutes. Cell wall and α-FtsZ signal are shown as in Figure 2. Scale bar = 5 µm. (B) Complementation of *ftsA_R286W_* Δ*zipA::aph* with *zipA_WT_* in *trans* from pKG110 specifically restores Kil-mediated filamentation. Shown are representative IFM micrographs of cell wall (red) or α-FtsZ (green) signal from fixed cells grown under *kil*-inducing conditions for 40 minutes (1 mM IPTG) of the indicated genotypes in a WM1074 *ftsA_R286W_* Δ*zipA::aph* pRR48-*kil* background. Signal and scale as in (A). (C) SulA and MinCD activity do not require *zipA*. Representative DIC micrographs of live cells with indicated plasmids in a WM1074 *ftsA_R286W_* Δ*zipA::aph* background grown with (bottom panels) or without (top panels) the indicated inducers for 40 min. Scale as in (A).

The *ftsA_R286W_* (ftsA*) gain of function mutation can bypass the loss of several normally essential cell division proteins, including ZipA or FtsK, as well as partially suppress an *ftsQ ts* mutant [49], [50], [51], [52]. This ability to stabilize the divisome prompted us to ask whether *ftsA_R286W_* cells might be more resistant to Kil. However, we found that *ftsA_R286W_* conferred no resistance to Kil produced from pRR48, as cells formed extensive filaments (data not shown). As a control, we also tested a *ftsA_R286W_* Δ*zipA::aph* double mutant for resistance to Kil, expecting similar results. Strikingly, these double mutant cells were entirely resistant to *kil* expression from pRR48, manifesting neither filamentation nor a loss of FtsZ ring localization following induction (Figure 6A). This strain background was also resistant to induction of *kil* from pBAD33 (data not shown). These results suggested that the absence of *zipA*, not the presence of *ftsA_R286W_*, imparts resistance to Kil. Note that *zipA^+^ ftsA_R286W_* cells are slightly shorter, while Δ*zipA ftsA_R286W_* cells are slightly longer than their wild-type counterparts [49].

To verify that the observed *kil*-resistance is specifically caused by an absence of *zipA*, we added the gene back in *trans* by introducing the compatible plasmid pKG110-*zipA* into the Δ*zipA ftsA_R286W_* strain containing pRR48-*kil*. Uninduced levels of *zipA* expressed from pKG110 in these cells did not perturb cell division on their own but were sufficient to restore *kil*-sensitivity (Figure 6B). Cells with the empty vector or expressing another early division protein, *zapA* [53], [54], [55], did not restore Kil-sensitivity in this Δ*zipA ftsA_R286W_* strain (Figure 6B). Unlike the isolated Kil-resistant *ftsZ* alleles, Δ*zipA ftsA_R286W_* cells remained sensitive to both SulA and MinC inhibition (Figure 6C), arguing that the Kil resistance of the Δ*zipA ftsA_R286W_* strain is not a general effect on FtsZ. Taken together, these results strongly suggest that ZipA is specifically required for the effect of Kil on FtsZ assembly and subsequently cell division.

### Additional Kil-resistant isolates map to *zipA*

**Figure 7:**
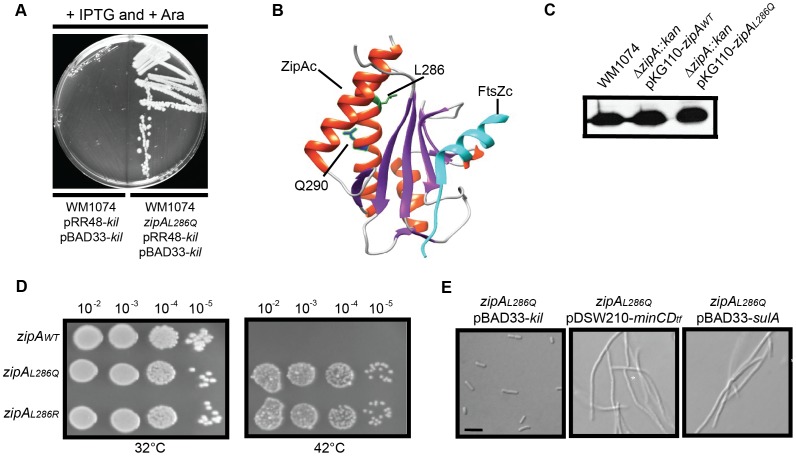
Isolation and characterization of Kil-resistant *zipA* alleles. (A) The *zipA_L286Q_* allele is a spontaneously isolated mutant resistant to loss of viability caused by *kil* expression from dual plasmids. Shown is an image of an LB plate with 1 mM IPTG and 0.2% arabinose to induce *kil* from pRR48 and pBAD33 simultaneously, with the original strain (left) and the *kil*-resistant isolate (right). Targeted sequencing of the *zipA* locus identified the resistant mutation as *zipA_L286Q_*. (B) Location of L286 and Q290 of ZipA in the co-crystal structure of the *E. coli* ZipA C-terminal domain (ZipA_C_) with an *E. coli* C-terminal FtsZ peptide (FtsZ_C_) [58], as generated by Chimera (http://www.cgl.ucsf.edu/chimera/). For ZipA_C_, α-helices are colored red and β-sheets are colored purple. The L286 sidechain is indicated in green and the Q290 sidechain in blue. FtsZ_C_ is colored cyan. (C) ZipA_WT_ and ZipA_L286Q_ expressed from pKG110 are made at levels comparable to native ZipA. Shown is an immunoblot against ZipA (upper panels) from whole cell extracts of indicated strains harvested during mid-log growth in the presence of 0.5 µM sodium salicylate. (D) Replacement of the native *zipA* gene with *zipA_L286Q_* or *zipA_L286R_* by recombineering confers Kil resistance. Spot dilutions of indicated *zipA* alleles in a CC4506 background strain are shown under conditions where *kil* is not expressed (32°C, left) and expressed (42°C, right). (E) Cells harboring *zipA_L286Q_* are resistant to Kil specifically, and not to MinCD or SulA. Representative DIC micrographs of WM1074 cells with the indicated plasmids are shown under inducing conditions (0.2% arabinose for pBAD33; 1 mM IPTG for pDSW210). Scale bar = 5 µm.

If ZipA were required for Kil-mediated inhibition of FtsZ as inferred from the above genetic experiments, we reasoned that it should be possible to isolate mutations in the essential *zipA* gene that confer Kil-resistance in a wild-type *ftsA* background without abrogating ZipA's normal function in the divisome. The powerful phenotype of Kil-induced cell filamentation and death allowed us to isolate spontaneous Kil-resistant mutants. To discourage the isolation of mutations that simply reduce *kil* expression or inactivate the *kil* product itself, we constructed a ‘double *kil*’ strain harboring both pBAD33-*kil* and pRR48-*kil* in WM1074. We isolated spontaneous Kil-resistant colonies by plating cultures of this strain on LB agar with both IPTG and arabinose (Figure 7A). Several independent isolates were saved and their *zipA* loci amplified for DNA sequencing.

All isolated *kil* resistant strains contained one of two mutations in *zipA*, encoding ZipA_L286Q_ and ZipA_L286R_, respectively. The C-terminal domain of ZipA containing this residue (ZipA_C_) directly interacts with the C terminus of FtsZ [56], [57], [58] (Figure 7B). As both alleles seemed to have similar properties, we chose ZipA_L286Q_ for further investigation.

To assess the general activity of ZipA_L286Q_ in cell division, we first cloned it into pKG110 and transformed the resulting plasmid into a WM1074 wild-type background. We then introduced the Δ*zipA::aph* allele by P1 transduction. The transduction efficiencies into cells harboring pKG110-*zipA_L286Q_* or pKG110-*zipA_WT_* were similar, verifying that ZipA_L286Q_ functions normally in cell division in place of the essential ZipA_WT_. Low-level induction of either *zipA_WT_* or *zipA_L286Q_* from pKG110 (with 0.5 µM sodium salicylate) in the WM1074 Δ*zipA::aph* background resulted in cellular levels of ZipA comparable to those in wild-type cells (Figure 7C) and caused no discernable differences in growth or division phenotypes. Additionally, overproduction of ZipA_WT_ [59] or ZipA_L286Q_ caused cell filamentation with multiple FtsZ rings present (data not shown).

To verify the Kil-resistance of *zipA_L286Q_*, we replaced the *zipA_WT_* gene of the W3110 strain harboring *kil* under *P_L_* control with *zipA_L286Q_* (or *zipA_L286R_*) by recombineering. The resulting strains displayed normal cell division phenotypes and resistance to *kil* upon derepression of promoter at 42°C (Figure 7D and data not shown). Finally, a fresh WM1074 wild-type background transduced with *zipA_L286Q_* linked to a *ptsH<>tet* marker was resistant to *kil* expression from pRR48, but remained sensitive to both SulA and MinCD inhibition (Figure 7E), demonstrating that this *zipA* allele is specific for resistance to Kil. Finally, random mutagenesis of *zipA* by PCR generated a mutation in a nearby residue, Q290R, (Figure 7B) which conferred the same level of Kil resistance to cells as the L286 mutations (data not shown), further arguing for the importance of this region of ZipA for Kil activity *in vivo*.

### The *kil* gene encodes a protein that interacts with ZipA and FtsZ

Early research on the *kil* gene identified a putative and abundantly produced protein product [60]. However, subsequent research established that this protein was actually λ Ea10, and that *kil* is likely expressed at only low levels from the *P_L_* promoter of λ phage ([2] and see below). Initial attempts to identify, purify, or chemically synthesize the protein product of the *kil* gene failed, prompting us to investigate whether the *kil* gene truly encodes a protein. Previous work established that insertion of a stop codon within the 5′ portion of *kil* or disruption of its stop codon abolishes Kil activity ([6] and Figure 1), but did not definitely show whether the loss of activity was caused by changes at the protein level or the nucleotide alterations themselves.

**Figure 8:**
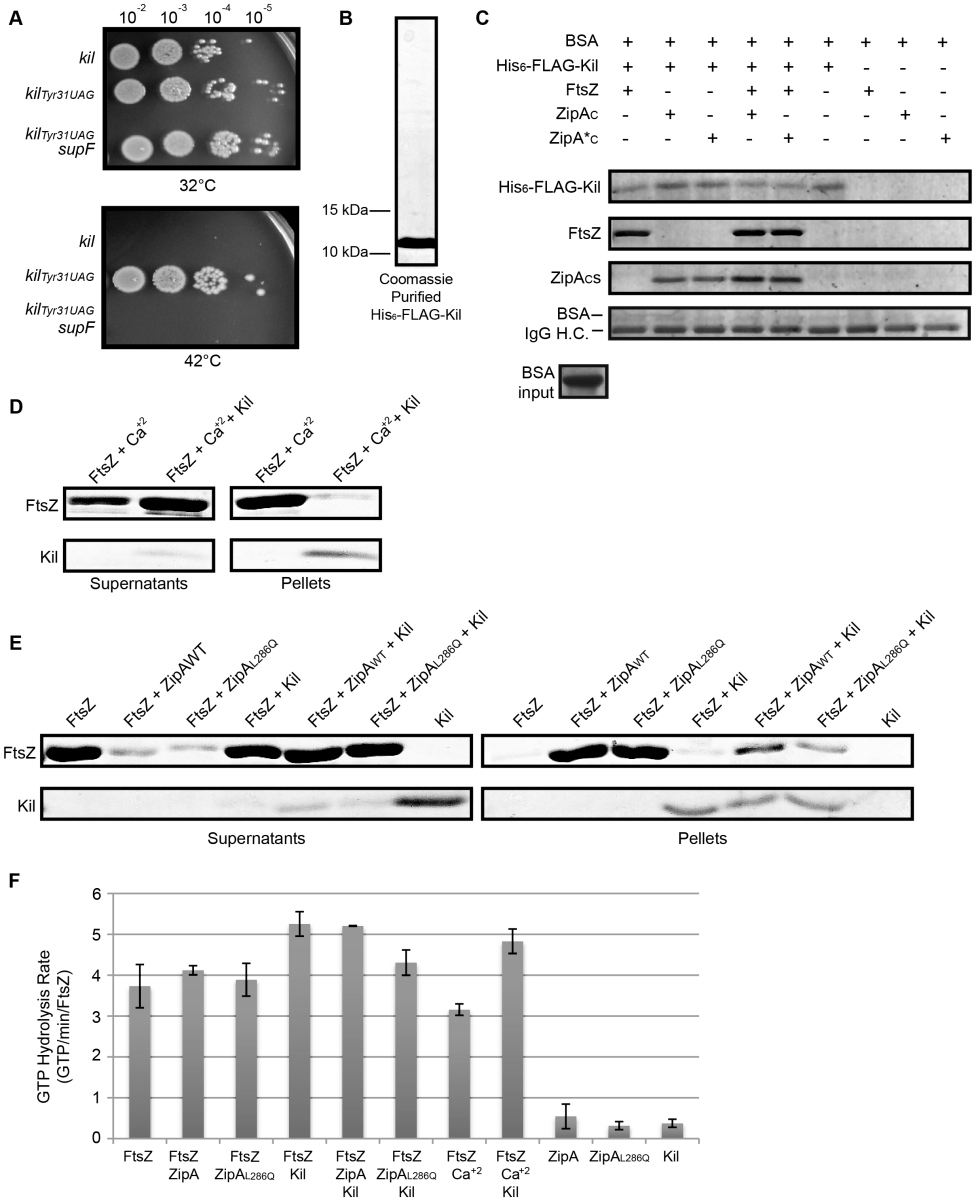
Kil protein copurifies with FtsZ and ZipA and inhibits FtsZ bundling *in vitro*. (A) The product of the *kil* gene is a protein. Spot dilutions incubated at 32 or 42°C of cells containing, under *P_L_* control, a wild type copy of *kil* (top row), a copy of *kil* where the thirty-first codon is replaced with an amber stop codon (middle row), or cells of an isogenic strain containing the *supF* gene that allows translation through the amber stop codon (bottom row). (B) His_6_-FLAG-Kil purifies as a single, intense band from BL21(DE3) *ftsA_R286W_ ΔzipA::aph* pBS58 cells. Shown is a Coomassie-stained 20% acrylamide SDS gel loaded with 15 µL purified His_6_-FLAG-Kil, with location of indicated size makers noted. (C) His_6_-FLAG-Kil interacts with FtsZ, ZipA_C_, and ZipA*_C_ in pull down assays. Various protein samples as indicated were incubated with α-FLAG resin and extensively washed, followed by SDS-PAGE and Coomassie staining. Proteins associated with His_6_-FLAG-Kil should be strongly enriched in the gels. Bovine serum albumin (BSA) was added to all input samples at the level shown and migrates immediately above the IgG heavy chain (IgG H.C.) in gels after pull downs (bottom strip). (D) Kil inhibits *in vitro* FtsZ assembly as assayed by sedimentation of calcium-bundled FtsZ polymer bundles. FtsZ and His_6_-FLAG-Kil bands are shown from Coomassie-stained gels of supernatant and pellet samples, following sedimentation of FtsZ assembly reactions in the presence of 1 mM GTP and other indicated components. (E) Kil inhibits FtsZ bundling by purified ZipA_WT_ or ZipA_L286Q_ C-terminal domains (ZipA_C_ or ZipA*_C_). Shown are FtsZ and His_6_-FLAG-Kil bands from Coomassie-stained gels of supernatant and pellet samples as in (D). (F) Kil slightly increases the GTPase activity of FtsZ. Rates of GTP hydrolysis are expressed as average GTP/min/FtsZ for the indicated combination of purified components in the presence of 1 mM GTP as in the sedimentation reactions in (D). Kil refers to His_6_-FLAG-Kil and ZipA or ZipA_L286Q_ refer to His-tagged C-terminal domains. Error bars represent the range of values between two replicate experiments.

To test this definitively, we inserted an amber stop mutation into the thirty-first codon (normally encoding tyrosine) of *kil* (*kil_tyr31UAG_*) to terminate translation prematurely. Cells harboring the *kil_tyr31UAG_ allele* under *P_L_* control survived at 42°C. To determine if cell survival was due to interrupted translation or due to a mutation in the RNA, we introduced the *supF* gene, which encodes a suppressor tRNA that would supply the originally encoded tyrosine amino acid at the amber mutation. The presence of *supF* restored Kil activity, confirming the product of *kil* functions as a protein (Figure 8A).

To obtain purified Kil protein, we cloned the *kil* gene into a pET vector, adding an N-terminally encoded FLAG-tag to create a *his_6_-flag-kil* fusion for expression and protein purification by cobalt-affinity chromatography. Purification of His_6_-FLAG-Kil yielded a single prominent band on Coomassie-stained SDS polyacrylamide gels that migrates near its predicted ∼10.5 kDa molecular weight with an estimated purity of >95% (Figure 8B). Induction of *his_6_-flag-kil* in BL21(DE3) cells inhibited cell division, indicating that the tagged fusion is active (data not shown). We found that optimal induction occurred in the absence of *zipA* and in the presence of extra FtsZ; we therefore preferentially expressed *his_6_-flag-kil* in a BL21(DE3) *ftsA_R286W_ ΔzipA::aph* pBS58 background.

Surprisingly, *his_6_-flag-kil* expression seemed to augment Kil activity *in vivo*, blocking cell division even in the absence of *zipA* (data not shown). Notably, this phenotype occurs with all tested Kil fusion derivatives, and no instances of complete suppression of any fusions have been isolated to date. With no antibody against Kil, we cannot know if this effect is merely because Kil fusions permit higher protein levels compared to wild-type untagged Kil, or if this effect arises from the increased length/size of a tagged Kil fusion.

Nonetheless, because ZipA is required for the activity of normal, untagged Kil on FtsZ assembly *in vivo*, we investigated whether Kil interacts directly with ZipA or FtsZ. We incubated purified, renatured His_6_-FLAG-Kil (see Materials and Methods) in the presence of α-FLAG M2 affinity resin with or without purified FtsZ and/or purified, His-tagged ZipA C-terminal domains (denoted ZipA_C_ for wild type *zipA* and ZipA*_C_ for *zipA_L286Q_*). Following binding of the resin by His_6_-FLAG-Kil, samples were washed extensively and retained proteins bound to the resin and/or His_6_-FLAG-Kil were visualized on Coomassie-stained SDS-polyacrylamide gels (Figure 8C). As expected, His_6_-FLAG-Kil remained bound to the resin following the washes, whereas FtsZ, ZipA_C_, or ZipA*_C_ were not resin-associated when incubated on their own without any His_6_-FLAG-Kil. However, FtsZ and each ZipA_C_ remained in samples following washes in the presence of His_6_-FLAG-Kil, arguing in favor of direct specific interactions between His_6_-FLAG-Kil and FtsZ, and between His_6_-FLAG-Kil and either ZipA_C_. In support of this, bovine serum albumin added to all the input mixtures at concentrations similar to those of FtsZ and ZipAc showed no specificity for the resin. Notably, His_6_-FLAG-Kil – ZipA_C_ interacts similarly with wild-type ZipA_C_ and the mutant ZipA*_C_, and the His_6_-FLAG-Kil – FtsZ interaction does not seem to be significantly augmented by either ZipA_C_ under the conditions of this experiment. Together these data suggest that Kil is capable of interacting independently with either FtsZ or the C-terminal domain of ZipA, and the L286Q mutation in ZipA does not prevent it from interacting with His_6_-FLAG-Kil.

### Purified Kil inhibits FtsZ bundling *in vitro*

Our *in vivo* evidence suggests that Kil inhibits FtsZ assembly, and that this activity normally requires the presence of ZipA. To test whether Kil could directly act to inhibit FtsZ assembly, we used sedimentation assays with purified FtsZ and His_6_-FLAG-Kil purified from the BL21(DE3) *ftsA_R286W_* Δ*zipA::aph* pBS58 background to ensure that little ZipA would be present in reactions. Not surprisingly, purified FtsZ does contain a small amount of copurified ZipA (data not shown), barely detectable by immunoblotting and not visible at the FtsZ concentrations used for sedimentation reactions.

We initiated FtsZ assembly with the addition of 1 mM GTP and then sedimented polymer bundles of FtsZ by centrifugation. Sedimentation primarily detects polymer bundles of FtsZ, and in our buffer conditions; little FtsZ sedimented in the presence of GTP alone, consistent with the relatively poor bundling capability of *E. coli* FtsZ [61]. Addition of calcium increased FtsZ polymer bundling and thus sedimentation, as previously reported [62], [63]. Strikingly, addition of His_6_-FLAG-Kil to FtsZ assembly reactions greatly reduced the amount of polymer sedimentation in the presence of calcium (Figure 8D), suggesting that the purified tagged Kil can directly inhibit FtsZ assembly *in vitro*.

To address the potential contribution of ZipA to Kil activity on FtsZ, we added purified ZipA_C_ or ZipA*_C_ to additional sedimentation reactions. As previously reported [56], ZipA_C_ increased polymer bundling, measured as sedimentation, similar to the effect of calcium. ZipA*_C_ also enhanced FtsZ sedimentation to similar levels. As with calcium, addition of His_6_-FLAG-Kil to the assembly reactions greatly reduced polymer sedimentation in the presence of either ZipA_C_ or ZipA*_C_ (Figure 8E). His_6_-FLAG-Kil co-sedimented with the small fraction of FtsZ polymer bundles that assembled in its presence, either with calcium (Figure 8D) or the ZipA_C_ domains, but did not sediment on its own (Figure 8E). This suggests that Kil may multimerize in the presence of FtsZ and/or form stable Kil-FtsZ complexes. Together, these data demonstrate that His_6_-FLAG-Kil interacts with FtsZ and inhibits its assembly *in vitro*, and suggest that the normal importance of ZipA *in vivo* for this activity is bypassed by the *in vitro* conditions.

To test the effect of Kil on FtsZ GTP hydrolysis rates, we performed GTPase activity assays under conditions identical to the sedimentation assays. GTP hydrolysis by FtsZ is induced by monomer-monomer interactions within a newly formed protofilament. If Kil inhibits FtsZ assembly by sequestering monomers, similar to SulA, it should strongly inhibit FtsZ GTP hydrolysis activity. If instead Kil severs FtsZ protofilaments or decreases FtsZ polymer bundling, this would increase the pools of monomers available to make new protofilaments, which should increase GTP hydrolysis.

The background GTPase activity signal from His_6_-FLAG-Kil, ZipA_C_, or ZipA*_C_ was negligible (∼0.5 GTP/min/FtsZ), and FtsZ displayed a rate of ∼3.2–4.1 GTP/min/FtsZ independent of bundling agent (Figure 8F). The presence of Kil in assembly reactions caused a small but significant increase in FtsZ GTP hydrolysis to 4.3–5.3 GTP/min/FtsZ. The effect of Kil was most pronounced in the presence of calcium (a 1.4-fold increase) and least pronounced in the presence of ZipA*_C_ (Figure 8F). Though modest, the across-the-board increase in GTP hydrolysis in the presence of Kil is consistent with either inhibition of FtsZ protofilament extension, severing protofilaments, or inhibition of FtsZ protofilament bundling.

### Kil-mediated inhibition of host cell division during phage infection

Our data inspire a model in which Kil interacts with FtsZ in a ZipA-dependent manner *in vivo* to inhibit FtsZ ring assembly and cause cell filamentation. The location of *kil* in the λ genome (Figure 1A) suggests that it would be expressed transiently during initial establishment of lysogeny and then upon exit of lysogeny when entering the lytic cycle. Normally, induction of a λ lysogen leads to rapid host cell lysis, but the potential role and importance of *kil* in this process is unknown. We therefore chose to investigate *kil* in its native context in intact *E. coli* λ lysogens, with the *cI857* allele to facilitate universal λ lysogen activation.

**Figure 9:**
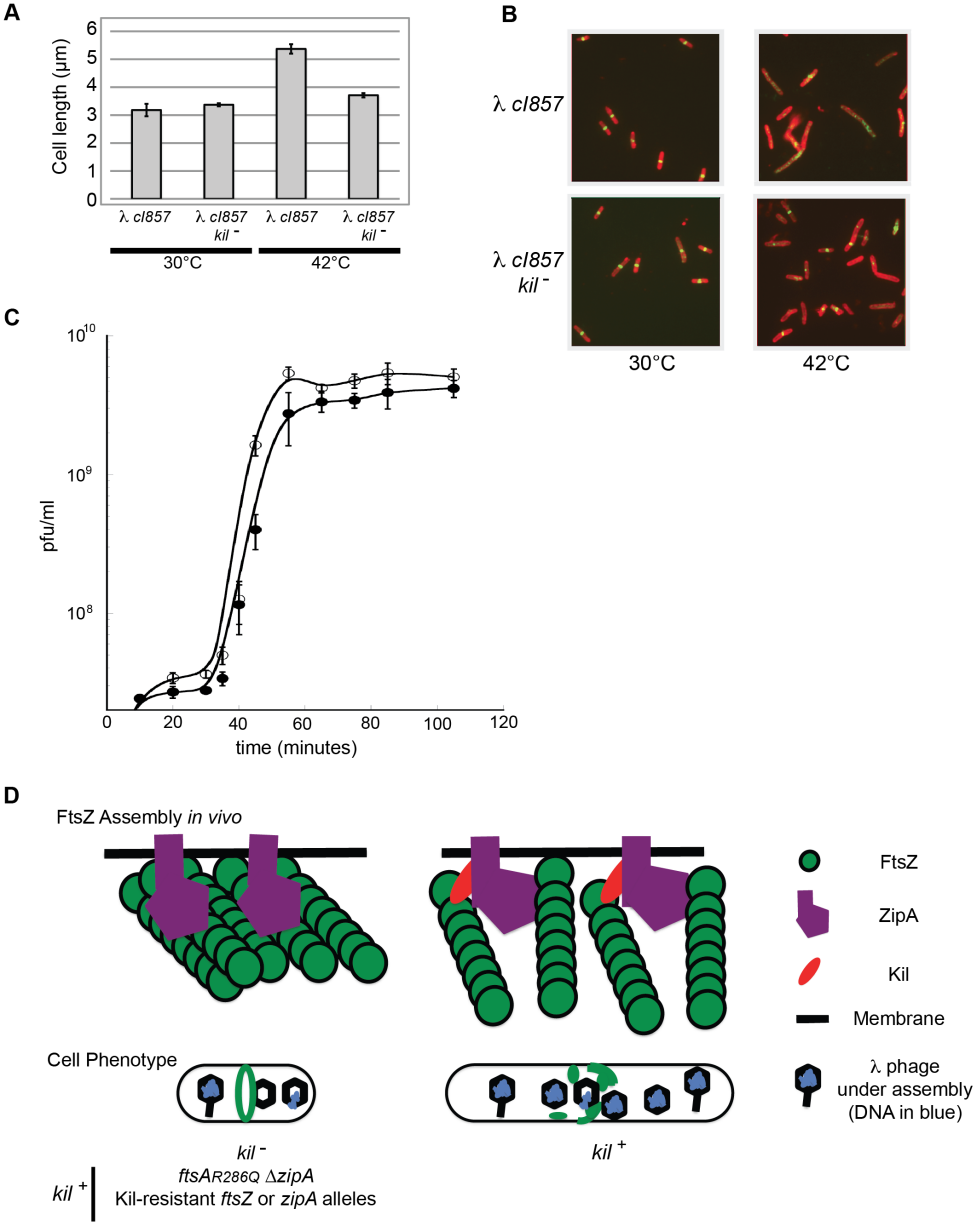
λ lysogen induction inhibits host cell division in a *kil*-dependent manner. (A) Induction of a λ lysogen results in a *kil*-dependent increase in *E. coli* cell length prior to host cell lysis. The average fixed-cell lengths of indicated *kil^+^* and *kil^−^* strain populations 40 minutes post λ lysogen induction (42°C), or control non-inducing conditions (30°C) are plotted. Error bars represent standard deviation between three separate population measurements. (B) Induction of a λ lysogen results in a *kil*-dependent, near-total ablation of FtsZ rings prior to host lysis. Representative IFM micrographs stained for cell wall (red) and α-FtsZ (green) are shown from the same fixed cells used for (A); the indicated genotypes and growth temperatures are listed. Cell wall and α-FtsZ signals are as described in Figure 2. Scale bar = 5 µm. (C) A one-step growth curve of *kil^+^* phage (solid circles) and *kil^−^* phage (open circles) showing plaque forming units (pfu) per mL over time in MG1655 at 39°C. (D) A model (with key to shapes on right) for Kil activity on FtsZ ring formation (top cartoons) is depicted for the indicated strain genotypes, and the resulting effects on cellular phenotype (lower cartoons). The top left cartoon illustrates the *kil^−^* condition alone, not all Kil-resistant conditions. The recruitment of Kil to midcell inhibits FtsZ assembly by completely blocking normal FtsZ ring assembly. FtsZ blobs and spirals seen by IFM suggest that FtsZ assembles aberrantly in the presence of Kil *in vivo*, unable to resolve into a closed ring structure. See discussion for the potential role of ZipA. Cell filamentation may increase lysis time in an un-compartmentalized host.

Induction of a λ lysogen by a shift to 42°C led to an increase in cell length up to 40 minutes post-induction (Figure 9A), at which time cells began to lyse. This increase in cell length is similar to that observed upon similar *P_L_* derepression in a defective prophage (Figure 2A), but does not proceed as rapidly or reach the point of extreme filamentation seen during *kil* expression. Induction of the λ lysogen also led to a rapid loss of FtsZ rings (Figure 9B).

The observed increase in cell length and rapid, complete loss of FtsZ ring assembly following induction of the λ lysogen did not occur in an isogenic *kil^−^* mutant. Although *kil^−^* cells still entered the lytic cycle with no observable delay, cell length did not increase significantly in the absence of *kil* (Figure 9A). Although FtsZ ring formation was partially inhibited under these conditions, perhaps by other λ components or through indirect effects on host cell physiology, the majority of *kil^−^* cells still contained FtsZ rings sufficient to maintain division (Figure 9B). These data indicate that *kil* expression has a dramatic effect on FtsZ ring formation, and subsequently on cell length, during the induction of a λ lysogen into the lytic cycle.

Finally, to ask whether *kil^−^* phage are compromised for reproduction, we monitored a single cycle of phage growth of *c*I*857* and a nonpolar deletion of *kil*, *c*I*857kil*Δ*7*, in MG1655 at 39°C (Figure 9C). Overall phage yields were not significantly affected by the absence of *kil*, although we observed a reproducibly shorter lysis time for the *c*I*857 kil*Δ*7* phage relative to *kil*. Kil effects on host cell lysis have been previously reported [7], [64].

## Discussion

The results reported here establish that the Kil protein of bacteriophage λ blocks host cell division by rapidly ablating FtsZ rings. In reactions with FtsZ purified from ZipA-deficient cells, purified tagged Kil prevents sedimentation of FtsZ bundled either by calcium or purified ZipA_C_. In addition, purified tagged Kil interacts with FtsZ in the presence or absence of ZipA. Together, these results suggest that Kil can interact directly with FtsZ *in vitro* to disrupt FtsZ protofilament bundling. However, ZipA is required for wild-type Kil to disrupt FtsZ rings normally *in vivo*, and point mutations in either FtsZ or ZipA can effectively resist FtsZ ring disruption by Kil. Excess FtsZ can also protect against inhibition by Kil *in vivo*, presumably by enhancing protofilament assembly and/or titrating out the inhibitor.

### A model for Kil-mediated inhibition of FtsZ ring assembly

The normal involvement of ZipA *in vivo* suggests that Kil may interact with both FtsZ and ZipA in the cell, a possibility supported by our *in vitro* interaction data. One explanation for the *in vivo* ZipA requirement is that ZipA may act specifically to recruit Kil to FtsZ protofilaments (Fig. 9D). The location of the mutations in ZipA that confer resistance to Kil is consistent with this idea. Although these mutations are in the FtsZ-binding domain of ZipA, the mutated L286 residue is not predicted to interact directly with the C-terminus of FtsZ, facing away from it in the co-crystal structure [58]. The L286 mutations could have an allosteric effect on ZipA-FtsZ interactions, but cells carrying *zipA_L286Q_* divide normally, so these interactions cannot be grossly altered. Moreover, over-expression of *zipA_L286Q_* causes cell division defects similar to that seen with *zipA_WT_* (data not shown), further arguing that the mutant is not dysfunctional in its effects on FtsZ assembly.

According to this model, ZipA_L286Q_ would be less able to recruit Kil to midcell than ZipA_WT_, presumably because of decreased ZipA-Kil interactions. However, our *in vitro* interaction data suggest no difference between His_6_-FLAG-Kil interaction with ZipA_C_ or ZipA*_C_. This disparity could stem from the additional length/size of the His_6_-FLAG-Kil fusion (that seems to act independently of ZipA *in vivo* unlike untagged Kil), or from the absence of full-length ZipA in the *in vitro* experiments. Additionally, results from the sedimentation experiment suggest that the high concentrations of Kil present in reactions might cause it to multimerize in the presence of FtsZ. Further work is required to clarify the precise mechanism of Kil activity and the role of ZipA in this activity.

The proposed ZipA-mediated recruitment of Kil to FtsZ *in vivo* is reminiscent of mechanisms proposed for other FtsZ inhibitors. For example, the cryptic prophage-derived DicB protein uses ZipA to recruit the DicB/MinC complex to the membrane-associated FtsZ ring [65]. However, unlike DicB, Kil inhibits FtsZ directly and does not require MinC for its inhibitory activity (Figure 3). The *B. subtilis* EzrA protein is related to ZipA in that it binds FtsZ and shares ZipA's unusual topology of N-terminal integral membrane domain and C-terminal cytoplasmic domain. A mutant of EzrA lacking this N-terminal transmembrane domain is able to inhibit FtsZ assembly directly *in vitro*, but *in vivo* this mutant does not localize to the membrane, thus lowering the local concentration of EzrA to a point where its interaction with FtsZ is no longer inhibitory [66]. By analogy, ZipA might serve to concentrate Kil near the membrane where FtsZ is located, enhancing its inhibition of FtsZ assembly (a function no longer needed *in vitro*). Kil is predominantly hydrophobic, and although it is not predicted to contain any transmembrane segment, it is predicted to have peripheral membrane association [67]. If this is the case, interactions with ZipA could still enhance localization of Kil specifically to midcell and/or position Kil optimally to interfere with FtsZ assembly within the *in vivo* environment that is absent in our *in vitro* FtsZ assembly assays.

Normal FtsZ assembly dynamics ensure that FtsZ rings properly form both temporally and spatially to link cell division with other cell cycle events [12]. Kil acts independently of the well-characterized host-derived systems of regulation, but may act on FtsZ assembly in a generally similar fashion. In support of this notion, our two Kil-resistant *ftsZ* alleles, *ftsZ_V208A_* and *ftsZ_L169R_*, display a general resistance to multiple forms of FtsZ assembly inhibition, and *ftsZ* alleles previously reported for SulA and MinC-resistance [46], [47], [48] also show similar degrees of resistance to Kil. Despite having lesions in the conserved T7-loop and near the nucleotide-binding region, these two mutant FtsZs are capable of FtsZ ring assembly *in vivo*, although many rings form at potential division sites other than midcell, including cell poles. This is consistent with a bias away from disassembly and towards stabilization. Such a bias would lead to resistance to disassembly factors such as MinC, even if Kil inhibits by a different mechanism. This resistance is incomplete, because Kil produced from a plasmid rapidly ablates FtsZ_V208A_ and FtsZ_L169R_ rings, and even Kil produced from the *P_L_* operon eventually overwhelms their ability to form rings and encourages acquisition of suppressors.

Recent publication of a crystal structure for a GDP-bound protofilament of FtsZ from *Mycobacterium tuberculosis* implicates residues adjacent to those corresponding to V208 and L169 of *E. coli* FtsZ as being involved in conformational changes at the subunit interface between straight, GTP-bound filament and curved, GDP-bound filament forms [45]. The general resistance of the FtsZ_V208A_ and FtsZ_L169R_ mutants to disassembly factors suggests that they may favor the GTP-bound form and have reduced GTP hydrolysis. The properties of other inhibitor-resistant FtsZ mutants fit this idea. The FtsZ9 mutant has extremely low GTPase activity and is highly resistant to SulA, MinC or Kil. In contrast, the FtsZ114 mutant, which has ∼50% of normal GTPase activity, confers only partial resistance to Kil or MinC [48]. The inhibitory activity of His_6_-FLAG-Kil on FtsZ filament bundling would be predicted to induce greater FtsZ subunit turnover and thus increase GTP hydrolysis, which is what we observe, and is similar to the effects of some other factors that inhibit FtsZ bundling such as EzrA [66], [68]. Consistent with Kil blocking FtsZ bundling, FtsZ localization in the presence of Kil becomes patchy and diffuse, possibly into broad helices, suggesting that FtsZ filaments still assemble, but fail to condense into a mature, bundled ring. An alternative explanation for the increased GTPase activity is that Kil may also prevent FtsZ protofilament elongation, which would increase the pool of free FtsZ monomers available to form dimers and oligomeric filaments capable of hydrolyzing GTP.

### Role of bacteriophage-encoded cell division inhibitors

A handful of other phage-encoded factors have been identified that block bacterial cell division, including a newly-described T7 gene product that also acts on FtsZ [69]. Other factors act through host-cell regulation of FtsZ assembly (DicB and *dicF* [28], [29]) or through an unknown mechanism (Kil of prophage Rac [26]). The lytic SPO1 phage of *Bacillus subtilis* encodes a peptide that inhibits host division prior to lysis [70], but acts at a stage following FtsZ ring formation (Haeusser and Margolin, unpublished data). Therefore, it seems that a variety of phage are able to target host cell division through unique peptides that act by diverse mechanisms. Suppression of host cell division by cryptic prophage-encoded factors has previously been implicated in increasing host adaptation to stress and resistance to antibiotics [71], and it is possible that *kil* benefits the host in some way during the lysogenic state.

In contrast to inhibiting FtsZ ring formation, a recent report described the role of *B. subtilis* Φ29 p1 in promoting phage replication through association with assembled FtsZ [72]. This strategy allows Φ29 to utilize an existing host cell scaffold to organize and optimize viral DNA production, but how a phage would benefit from perturbing FtsZ ring formation and inhibiting cell division is less clear. The original studies on *kil^−^* λ phage suggested that they still replicate, assemble, and lyse the host comparably to *kil^+^* counterparts, with similar burst sizes [3], [6], [73]. However, these studies used *c*III*67* mutant phage, which may be more prone to lysogeny and mask a *kil^−^* growth phenotype. Here, we show that Kil activity is evident upon lytic induction of λ, and demonstrate that, in a single cycle of growth, the *kil^−^* mutant phage has a shorter latent period and causes earlier cell lysis. Although we did not observe a decrease in yield for the *kil* mutant phage, in some circumstances a shorter latent period may result in lower phage yieldl. The larger average volume of *kil^+^* cells relative to *kil^−^* cells might translate into a longer time to induce lysis. Cell size changes may also affect the lysis-lysogeny decision [74].

ln addition to affecting the timing of lysis, *kil* could benefit phage by creating a non-compartmentalized host that permits easier and more accurate phage reproduction. In this scenario, activation of cell division and construction of a septum could interfere with excision of the λ lysogen, its replication, or its packaging. Previous studies with *kil* from the lambdoid P22 phage of *Salmonella* found that *kil* expression was required for efficient lytic growth of *abc^−^* mutants lacking anti-RecBCD activity, increasing burst size by eight fold [75]. This suggests Kil activity becomes important during conditions of high recombination frequency. By analogy, *B. subtilis* Maf induces a temporary block in cell division during natural competence development that permits efficient, uninterrupted DNA recombination [76].

The lack of an obvious *kil^−^* phenotype for λ phage in original studies [3] and the subtle defects reported here could arise from biased, unnatural laboratory growth conditions, or an overlapping function with other phage components, such as *gam*. The abrupt but temporary loss of FtsZ rings upon *P_L_* derepression in the absence of *kil* and the relatively large percentage of cells that lose FtsZ ring formation even in an otherwise complete *kil^−^* λ lysogen suggest that Kil may not act completely independently. Future experiments will focus on clarifying the molecular mechanism of Kil-mediated FtsZ ring disruption and uncovering its role in λ phage biology.

## Materials and Methods

### Strains and growth conditions

All *E. coli* strains used are listed in Table 1. Standard genetic methods including transformation and P1 *vir* transduction were used for strain construction. Recombineering methods for strain construction [77], [78] are described in a section below.

**Table 1:** Strains used in this study.

Strain	Description	Source
W3110	*rpoS_am_ rph-1* INV(*rrnD-rrnE*)	Lab strain
WM1074	TX3772 (MG1655: *ilvG rpb-50 rph-1* Δ*lacU169*)	Lab strain
C600	*thi-1 thr-1 leuB6 lacY1 tonA21 supE44*(*glnV44*) *rfbC1 fhuA1*	Lab strain
DY330	W3110 Δ*lacU169 gal490* λ[*cI857* Δ*(cro-bioA)*]	[93]
CC4506	DY330 λ[*(sieB-ea10)<>cat cIII^−^ kil^+^ (gamL-int)<>tet*]	[6]
CC4512	DY330 λ[*(sieB-ea10)<>cat cIII^−^ kil^−^ (gamL-int)<>tet*]	[6]
JW0941	BW25113 *ΔsulA773::kan*	[94]
CH5	Strain harboring Δ*zipA::aph* allele	[59]
NB4	CC4506 [pSEB162]	This work
NB5	CC4506 [pSEB25]	This work
NB6	CC4506 [pJPB71]	This work
NB7	CC4506 [pJPB223]	This work
NB8	CC4506 [pBR322]	This work
NB9	CC4506 [pJP10]	This work
NB15	CC4506 [pNB15]	This work
AW34	W3110 *ftsZ84 gal490 lacΔU169 leuD::*Tn*10 mutS<>amp* λ[*int::lacZ<amber>kil cI857 Δ[cro-bio]* [pSIM18]	This work
AW41	DY330 λ[*int::lacZ (sieB-ea10)<>cat cI857 Δ*[*cro-bio*]] [pSIM18]	This work
AW59	AW41 *leuD*::Tn*10 ftsZ_V208A_*	This work
AW60	AW41 *leuD*::Tn*10 ftsZ_L169R_* [pSIM18]	This work
AW64	AW41 *leuO*<>*tetA* [pSIM18]	This work
AW65	AW64 *mutS<>amp*	This work
LT447	MG1655 λ[*cI857*]	Lab strain
LT1566	MG1655 λ[*cI857 kil::IS2*]	Lab strain
LT352	MG1655 *polA resA1*	Lab strain
MH22	AW65 *ftsZ_V208A_*	This work
MH23	AW65 *ftsZ_L169R_*	This work
MH37	AW41 *ptsH<>tetA mutS<>amp* [pSIM18]	This work
MH60	AW41 without pSIM18	This work
MH79	MH60 *zipA*_L286Q_ *ptsH<>tetA*	This work
MH80	MH60 *zipA*_L286R_ *ptsH<>tetA*	This work
MH124	MH37 *zipA_Q290R_* (and lost pSIM18)	This work
MH112	MH113 with *trp*::*Tn*10 *supF*	This work
MH113	*mutS<>amp* λ[*int::lacZ<>kil_tyr31UAG_ (sieB-ea10)<>cat cI857 Δ*[*cro-bio*]]	This work
JS549	MG1665 *galK_TYR145UAG_* Δ*tyrTV*<>	This work
DPH65	WM1074 [pRR48_MCSpKG_-*kil*]	This work
DPH66	W3110 [pRR48_MCSpKG_-*kil*]	This work
DPH69	WM1074 [pRR48_MCSpKG_]	This work
DPH99	BL21(DE3) [pBS58]	This work
DPH208	WM1074 [pBAD33-*kil*]	This work
DPH209	W3110 [pBAD33-*kil*]	This work
DPH308	WM1032 [pBAD33-*kil*]	This work
DPH327	DPH538 [pBAD33-*sulA*]	This work
DPH538	WM1657 [pRR48_MCSpKG_-*kil*]	This work
DPH539	DPH538 [pKG110]	This work
DPH541	DPH538 [pKG110-*zipA*]	This work
DPH551	DPH538 [pKG110-*zapA*]	This work
DPH561	WM2775 [pBAD33-*kil*]	This work
DPH565	WM1659 [pRR48_MCSpKG_-*kil*]	This work
DPH625	DPH538 [pKG110-*zipA_L286Q_*]	This work
DPH629	WM1074 [pKG110-*zipA*]	This work
DPH630	WM1074 [pKG110-*zipA_L286Q_*]	This work
DPH638	AW59 [pRR48_MCSpKG_-*kil*]	This work
DPH639	AW60 [pRR48_MCSpKG_-*kil*]	This work
DPH640	AW64 [pRR48_MCSpKG_-*kil*]	This work
DPH643	DPH629 Δ*zipA::aph*	This work
DPH644	DPH630 Δ*zipA::aph*	This work
DPH647	BL21(DE3) [pET28a-*his*-zipA*_C_-his]	This work
DPH653	DPH99 [pET28a-*his-flag-kil*]	This work
DPH657	WM3052 [pBAD33-*kil*]	This work
DPH658	WM3053 [pBAD33-*kil*]	This work
DPH659	WM3054 [pBAD33-*kil*]	This work
DPH672	BL21(DE3) *ftsA_R286W_* (ftsA*) *sac284-Tn10* [pBS58]	This work
DPH673	DPH672 Δ*zipA::aph*	This work
DPH682	DPH673 [pET15a-*his-flag-kil*]	This work
DPH696	W3110 [pRR48_MCSpKG_]	This work
DPH698	WM1074 Δ*sulA::kan*	This work
DPH701	DPH698 [pBAD33-*kil*]	This work
DPH705	WM1074 [pBAD33]	This work
DPH710	WM1074 *zipA*_L286R_ *ptsH<>tetA*	This work
DPH711	DPH682 [pKG110-*zipA_L286Q_*]	This work
DPH714	AW59 [pDSW210-*his*-*minCD*]	This work
DPH715	AW60 [pDSW210-*his*-*minCD*]	This work
DPH716	AW64 [pDSW210-*his*-*minCD*]	This work
DPH717	AW59 [pBAD33-*sulA*]	This work
DPH718	AW60 [pBAD33-*sulA*]	This work
DPH719	AW64 [pBAD33-*sulA*]	This work
DPH722	WM1657 [pDSW210-*his-minCD*]	This work
DPH724	DPH710 [pDSW210-*his-minCD*]	This work
DPH725	DPH710 [pBAD33-*sulA*]	This work
DPH735	DPH710 [pBAD33-*kil*]	This work
WM971	BL21(DE3) [pET11a-*ftsZ*]	H. Erickson
WM1032	WM1074 *ΔminCDE::kan*	[95]
WM1614	BL21(DE3) [pET28a-*his*-*zipA_C_-his*]	This work
WM1657	WM1659 Δ*zipA::aph*	[49]
WM1659	WM1074 *ftsA_R286W_* (ftsA*)	[49]
WM2313	W3110 [pBAD33]	This work
WM2775	WM1074 Δ*ttk::kan* (Δ*slmA::kan*)	[50]
WM3052	PB143 *ftsZ^−^ recA::Tn10* [pBEF0]	[46]
WM3053	PB143 *ftsZ^−^ recA::Tn10* [pBEF9]	[46]
WM3054	PB143 *ftsZ^−^ recA::Tn10* [pBEF114]	[46]

Cells were grown in Luria-Bertani (LB) medium at 30°C or 32°C, as indicated, for temperature-sensitive (*ts*) strains under permissive conditions and 42°C under non-permissive conditions, or at 37°C for all non-*ts* strains. Optical density readings at 600 nm (OD_600_) were measured using a UV-1601 or UV-1800 spectrophotometer (Shimadzu). LB medium was supplemented with ampicillin (50 µg/ml; Fisher Scientific), kanamycin (50 µg/ml; Sigma-Aldrich), chloramphenicol (20 µg/ml; Acros Organics), tetracycline (10 µg/ml; Sigma-Aldrich), and spectinomycin (100 µg/ml; Sigma-Aldrich), as needed. Gene expression from vectors derived from pET28-, pET15- (Novagen – EMD Millipore), and pRR48 [79] was induced with 1 mM isopropyl-β-D-galactopyranoside (IPTG) (Fisher Scientific). Gene expression from pBAD33-derived vectors [80] was induced at a final concentration of 0.2% L-(+)-arabinose (Ara) (Sigma-Aldrich). Gene expression from pKG110-derived vectors (J.S. Parkinson, University of Utah) was either uninduced or induced with 0.5 µM sodium salicylate (Mallinkrodt), as indicated. A PCR test [81] confirmed that LT447 and LT1566 were monolysogens.

### DNA and protein manipulation and analysis

Standard protocols or manufacturers' instructions were used to isolate plasmid DNA, as well as for restriction endonuclease, DNA ligase, PCR, and other enzymatic treatments of plasmids and DNA fragments. Enzymes were purchased from New England BioLabs, Inc. (NEB) or Invitrogen. Plasmid DNA was prepared using the Wizard Plus SV Minipreps DNA Purification Kit, PCR and digest reactions were cleaned up using the Wizard SV Gel and PCR Clean-up System, and chromosomal DNA was prepared using the Wizard Genomic DNA Purification Kit (Promega). KAPA HiFi HotStart DNA polymerase (Kapa Biosystems) or Phusion High-Fidelity DNA polymerase (Thermo Scientific – NEB) or Platinum Taq (Invitrogen) were used for PCR reactions in a MyCycler Thermal Cycler (Bio-Rad). Oligonucleotides were purchased from Sigma-Aldrich or IDT and their sequences are listed in Table S1. The final versions of all cloning products were sequenced to verify their construction. DNA sequencing was performed by GeneWiz (South Plainfield, NJ), SeqWright (Houston, TX) or SAIC-Frederick, Inc. (Frederick, MD). DNA bands were visualized using an AlphaImager Mini System (ProteinSimple) and DNA concentrations were estimated with a NanoDrop ND-1000 Spectrophotometer (Thermo Scientific). Protein concentrations were determined using the Coomassie Plus Assay (Themo Scientific – Pierce). Molecular graphics and analyses were performed with the UCSF Chimera package (http://www.cgl.ucsf.edu/chimera). Chimera is developed by the Resource for Biocomputing, Visualization, and Informatics at the University of California, San Francisco (supported by NIGMS P41-GM103311).

### Plasmid construction

All plasmids are listed in Table 2.

**Table 2:** Plasmids used in this study.

Plasmid	Description	Source
pBAD33	pACYC184 derivative containing the *araBAD* promoter	[80]
pBR322	Cloning plasmid	NEB
pET15a	Overexpression plasmid	Novagen
pET28a	Overexpression plasmid	Novagen
pHDB3	pBR322 derivative	[84]
pKG110	pACYC184 derivative containing the *nahG* promoter	J. Parkinson
pRR48	pBR322 derivative with a weakened *lac* promoter	[79]
pSIM18	Hy^R^, pSC101 *repA_ts_* recombineering plasmid	[96]
pJPB71	pSC101-*ftsZ*	[31]
pJBP223	pSC101-*ftsQAZ*	[48]
pSEB25	pSC101-*ftsQZ*	[48]
pSEB162	pSC101-*ftsQA*	[48]
pBEF0	pGB2-derivative with wild-type *ftsZ*	[46]
pBEF9	pGB2-derivative with *ftsZ9* allele	[46]
pBEF114	pGB2-derivative with *ftsZ114* allele	[46]
pBS58	pGB2-derivative with *ftsQAZ*	[97]
pJP10	pHDB3 with ∼5.5 kb fragment including ‘*murC*-*ddlB*-*ftsQ*-*ftsA*-*ftsZ*-*lpxC*’	This work
pNB15	pBR322-*sdiA*	This work
pDH94	pRR48_MCSpKG_-*kil*	This work
pDH104	pBAD33-*kil*	This work
pDH139	pET28a-*his-flag-kil*	This work
pDH145	pKG110-*zipA_L286Q_*	This work
pDH146	pET28a-*his*-zipA*_C_-his (*zipA_L286_* C-terminal domain)	This work
pDH149	pET15a-*his-flag-kil*	This work
pWM971	pET11a-*ftsZ*	H. Erickson
pWM1610	pET28a-*his-zipA_C_-his*	This work
pWM1737	pBAD33-*sulA*	[98]
pDSW210	pBR322 derivative with a weakened *lac* promoter	[99]
pWM2737	pDSW210-*his*-*minC-minD* translational fusion (*minCD*)	This work
pWM3073	pKG110-*zipA*	[82]
pWM4647	pKG110-*zapA*	This work

Kil expression plasmids: For pRR48-*kil*, the *kil* coding sequence was amplified from CC4506 chromosomal DNA with primers DPH199 and 200; the PCR product was digested with *Pst*I and *Spe*I, then ligated into a pRR48-derivative where the original multiple cloning site had been replaced (*Nde*I to *Sal*I) with that of pKG110. For pDH104 (pBAD33-*kil*), the *kil* coding sequence with its native ribosome-binding site was amplified from CC4506 chromosomal DNA with primers DPH217 and 202; the PCR product was digested with *Xma*I and *Pst*I, then ligated into pBAD33.

Toxic overexpression of *minCD*: For pWM2737 (pDSW210-*his*-*minCD*) the *minD* coding sequence was amplified with primers WM960 & 356, the PCR product was digested with *Sal*I and *Hind*III, then ligated into pWM2735 (pDSW210-*his_6_-minC*) [44] to create sequence encoding an uninterrupted His-tagged, MinCD translational fusion (*minCD_tf_*).

Complementation of Δ*zipA* cells with the *zipA_L286Q_* allele: pDH145 (pKG110-*zipA_L286Q_*) was constructed as previously published for pKG110-*zipA* [82], but using DPH615 (*zipA_L286Q_*) chromosomal DNA as template. Plasmid pKG110-*zapA* was constructed by Daisuke Shiomi in the same manner as pKG116-*zapA* [82].

Plasmids for protein purifications: For pET28-*zipA_C_* the C-terminal ZipA domain coding sequence was amplified by Tushar Beuria with primers WM269 & 268; the PCR product was digested with *BamH*I and *Xho*I, then ligated into pET28a. As primer WM268 did not include a stop codon, this construct produces His_6_-ZipA_C_-His_6_. Construction of pDH146 (pET28-zipA*_C_) was therefore performed identically, but with DPH615 chromosomal DNA as template, to produce a double His-tagged version of ZipA_C_ that harbors the L286Q mutation. For pDH139 (pET28-*flag-kil*) the *kil* coding sequence with its stop codon was amplified from CC4506 chromosomal DNA in two steps (primers DPH309 & 170, followed by DPH310 & 170) to add sequence encoding a FLAG-tag to the 5′ end of *kil*, in-frame with the His-tag-encoding codons of the plasmid. The resulting product was digested with *BamH*I and *Hind*III, then ligated into pET28a. Plasmid pDH149 (pET15-*flag-kil*) was constructed by digesting pDH139 with *Nco*I and *Xho*I, then ligating the *his_6_-flag-kil* insert into identically-digested pET15b.

### General recombineering methods

Recombineering was done using published methods [77], [78]. Briefly, the Red functions were expressed either from the defective prophage or from a recombineering plasmid such as pSIM18, a hygromycin resistant plasmid encoding the temperature-sensitive CI857 repressor and a portion of the *P_L_* operon containing *gam*, *exo*, and *bet*. Log phase cells propagated at 32°C were subjected to a 15 minute heat pulse in a 42°C shaking water bath to induce the Red functions, quickly chilled in an ice-water slurry, and prepared for electroporation by washing. DNA, either double- or single-stranded, was introduced into the cells by electroporation. When a drug marker was selected, the cells were allowed to recover for several hours in 1 mL broth before plating. When point mutations were introduced with ∼70 base oligonucleotides, the cells were plated non-selectively on LB agar plates after a 30 minute recovery time, either at 42°C for direct selection of Kil-resistant alleles or at 32°C for screening by PCR, as described below. In some cases oligonucleotides with additional sequence changes in third positions (wobble positions) were used to create mutations. As described [83], these additional wobble changes were placed near the change of interest, creating a configuration of mispairs that is not recognized by the *E. coli* mismatch repair system, without changing the amino acid sequence. This allows both high-efficiency recombineering and detection of recombinant chromosomes with PCR. When wobble changes were introduced, between 12–20 colonies were analyzed, using one oligonucleotide that hybridized specifically to the recombinant sequence, paired with a second nearby oligonucleotide; this primer pair should not yield product with the parental sequence. Positive candidates were purified to single colonies and about a dozen of these single colonies were again subjected to the same PCR analysis. The region of interest from positive candidates identified in the second round of PCR screening was sequenced. Sequences for oligonucleotides used are listed in Table S1.

### Construction of a non-polar deletion of *kil* in λ *c*I*857* phage

A seven-codon in-frame deletion within *kil*, removing codons 20–26, was initially constructed in XTL241 (HME6 *gam<>cat-sacB*) and then moved to a phage. The deletion was built into a hybrid oligonucleotide, LT793, which along with an oligonucleotide to the downstream *gam* gene, LT795, were used to amplify a PCR product containing the deletion and spanning the region containing the counter-selectable marker. Recombineering was used to replace the *cat-sacB*. After introduction of the PCR product into electrocompetent XTL241 induced for the Red functions, cultures were grown overnight and recombinants were selected on sucrose plates at 32°C. Candidate colonies were purified on sucrose and confirmed to be chloramphenicol-sensitive. Primers AW24 and LT795 were used to amplify the region containing the deletion, which was confirmed by sequencing. In contrast to the *kil* ^+^ control, cells containing the deletion did not filament after several hours of growth at 42°C, and formed colonies after overnight growth at 42°C. A lysate of λ *c*I*857red3* was grown on cells containing the seven-codon deletion to allow marker rescue from the defective prophage onto the phage. The lysate was plated on LT352. Plaques were picked into 0.5 mL TMG (10 mM Tris base, 10 mM MgS0_4_ 0.01% gelatin) and 2 µl of the pickate was used for PCR with oligonucleotides AW24 and LT795: the difference in size between the deletion and the wild-type alleles was resolvable on a 1.2% agarose gel. The deletion phage was purified by another round of plating on LT352 and a high titer plate lysate was grown from a purified plaque on C600.

### Isolation of multicopy suppressors of *kil*

CC4506 was transformed with a pBR322-based library containing 1.5–5 kb *Sau*3A fragments of LE392 genomic DNA cloned into pHDB3 [84] and transformants were selected on LB ampicillin agar at 42°C. Ampicillin-resistant colonies that survived Kil induction were pooled, plasmid DNA was isolated and CC4506 was transformed with this enriched plasmid population to confirm suppression. The *Sau*3A library was kindly provided by Nadim Magdalani (NIH). Plasmid pJP10 was isolated by this procedure. Plasmid pNB15 was isolated by a similar procedure except the pBR322-based library was constructed by cloning 2–4 Kb *Sau*3A fragments of JS549 genomic DNA into pBR322 cut with *Bam*HI.

### Isolation of Kil-resistant *ftsZ* alleles

The *ftsZ* gene was amplified from W3110 with oligonucleotides XMZ325 and XMZ326, using standard PCR conditions. This *ftsZ* PCR product was used as a template for mutagenic PCR [85] with the same oligonucleotides. This randomly mutagenized pool of *ftsZ* PCR fragments was used for recombineering into strain AW34, which carries the thermo-sensitive *ftsZ84* allele (G105S) linked to *leuD::*Tn*10*, a defective λ prophage with thermo-inducible *kil* expression, and a *mutS<>amp* allele to prevent host mismatch repair during recombination. After a 30 minute recovery in broth at 30°C, aliquots were plated on nitrocellulose filters on LB plates and incubated at 32°C for 3 hours. Filters were then transferred to 42°C pre-warmed LB plates and these plates were subsequently incubated overnight at 42°C to select for growth. This procedure simultaneously selects for recombinants that replace *ftsZ84* with the wild type allele and for resistance to λ Kil expression. A lysate of P1 *vir* was grown on a pool of the temperature-resistant isolates. This lysate was used to transduce the Kil-expressing strain AW41, selecting tetracycline resistance at 42°C. Thermo-resistant colonies were purified and their *ftsZ* gene was amplified with colony PCR; the resulting PCR products were sequenced. Several isolates of the *ftsZ*_V208A_ mutation were obtained, and only one isolate of *ftsZ_L169R_*. One isolate of each of the mutant types was chosen for further characterization. The mutations were introduced into a fresh background by P1 transduction at 32°C, selecting for the linked *leuD<>*Tn*10* and screening for temperature resistance.

### Isolation of Kil-resistant *zipA* alleles

Competent WM1074 cells were sequentially transformed with pBAD33-*kil* and pRR48-*kil* and the resulting strain was verified to have a *kil^+^* phenotype upon induction of *kil* from either plasmid. A culture of this ‘double *kil*’ strain was grown to mid-logarithmic phase and induced with IPTG and arabinose simultaneously. 100 µL of stationary phase culture was plated on LB agar supplemented with appropriate antibiotics, IPTG, and arabinose and grown overnight at 37°C. All resulting colonies were purified, then grown in liquid media to freeze samples. Chromosome purification and PCR amplification of the *zipA* locus in the saved isolates showed that all contained a *zipA_L286Q_* mutation, while the original WM1074 pBAD33-*kil* pRR48-*kil* strain contained *zipA_WT_* sequence.

A second isolation of spontaneous Kil-resistant mutants was carried out using a similar protocol, with the only difference being that cells were grown in liquid culture under non-inducing conditions into stationary phase, then plated on medium containing IPTG and arabinose. All isolated colonies from this second experiment contained a *zipA_L286R_* mutation.

### Using ssDNA recombination to isolate, map and confirm suppressor mutations

Recombineering using ssDNA can be very efficient with up to 75% of the cells being recombinants [83]. Therefore, recombineering with ssDNA can be used to isolate and map mutations, and these techniques were used throughout this work. For example, when mutagenic PCR was used to isolate *zipA* mutations, in one case sequencing revealed a double mutation, *zipA_A245T,Q290R_*. We designed and ordered oligonucleotides to make each mutation separately via recombineering. Recombineering-proficient cells prepared on CC4506 were transformed with each oligonucleotide separately or both together, outgrown for 30 minutes, then diluted and spot-titered on LB plates at 30°C and 42°C. We found that only one of the oligonucleotides, MH82, was necessary and sufficient to create a mutation that suppressed Kil-dependent killing.

### Spot titers

Cells used for spot titers were taken from the same cultures used for fixation unless as noted below. A ten-fold dilution of these cultures was taken approximately forty minutes after induction (or control conditions), unless otherwise noted, and then serially diluted into fresh LB media in a 96-well plate using a multichannel pipette. A flame-sterilized and cooled, metal-pronged tool was then used to replica-plate spots of serially diluted culture onto LB plates with added components and incubation conditions as indicated in the text and figures. Photos of plates were taken in a FluoroChem 8800 system with its accompanying camera and software (Alpha Innotech).

Spot titers in Figures 4C and 7D were done as follows: An overnight culture was diluted 100-fold and cells were grown at 30° for 2 hours in LB. Ten-fold serial dilutions of these cultures were made in TMG (see above) and 10 µl was spotted on pre-warmed LB plates and incubated at the indicated temperatures.

### Determining loss of viability after exposure to Kil

To generate the data in Figure 1D, an overnight culture of CC4506 was diluted 70-fold into 15 mL of LB broth and grown to an OD_600_∼0.25 at 32°. The culture was diluted in 10-fold increments from 10^0^ to 10^−5^. At time “0”, 0.1 mL samples of appropriate dilutions were spread on prewarmed 42° LB plates on which we had placed a sterile 82 mm (diameter) nitrocellulose filter. After the indicated time, sterile forceps were used to move the filter to a 32° plate. These LB plates were incubated overnight at 32° and colonies counted.

### Cell fixation, microscopy, and analysis

Overnight cultures were started from −80°C strain stocks and grown under appropriate antibiotic selection and permissive conditions. Overnight cultures were diluted into fresh medium, grown to mid-logarithmic phase, then the OD_600_ of individual cultures was adjusted to a uniform OD_600_ = 0.025 with fresh medium. Cultures were grown for approximately 2 doublings at permissive conditions and then shifted to non-permissive conditions (or kept as permissive controls) at time point zero. Unless otherwise indicated, samples of culture for microscopy were taken approximately 40 minutes after shifting to non-permissive conditions.

Cells were fixed with methanol and processed for immunofluorescence microscopy (IFM) as previously published [86] using lysozyme (Sigma) treatment for 5 minutes and antibodies diluted in bovine serum albumin (Fisher Scientific). Primary polyclonal rabbit α-FtsZ [87] was used at 1/2500, secondary goat α-rabbit-AlexaFluor 488 (Molecular Probes) and wheat germ agglutinin conjugated to rhodamine (Molecular Probes), to visualize cell wall, were used at 1/200. For DNA staining, 4′,6-diamidino-2-phenylindole (DAPI) (Molecular Probes) was used at 0.5 µg/mL. Micrograph images were captured on an Olympus BX60 microsope with a Hamamatsu C8484 camera using HC Image software (Hamamatsu). Cell length and ring frequency measurements were taken with the ObjectJ extension [88] (http://simon.bio.uva.nl/objectj/) of ImageJ [89] (National Institutes of Health) using a minimum number of 100 cells and images were minimally adjusted for brightness/contrast using Adobe Photoshop CS4. Microsoft Excel 2008 for Mac (v. 12.3.6) was used for data tabulation and calculatio

### Immunoblot analysis

Samples were prepared, subjected to SDS-PAGE on 20% acrylamide gels, and transferred as published [90]. Transfer of His_6_-FLAG-Kil samples was done in 10 mM CAPS pH 10.0, 10% methanol transfer buffer with a two-hour transfer time. Mouse monoclonal α-His primary antibody (Sigma-Aldrich) and affinity-purified rabbit polyclonal α-FtsZ [87] were used at 1/5000. Affinity-purified rabbit polyclonal α-ZipA [49] was used at 1/1000. Goat α-mouse and α-rabbit secondary antibodies conjugated to horseradish peroxidase (HRP) (Sigma-Aldrich) were used at 1/10,000. A SuperSignal West Pico Chemiluminescent Substrate Kit (Thermo Scientific – Pierce) was used for HRP detection; blots were exposed to film and developed using a Konica SRX101A Film Processor (Konica Minolta).

### Protein purification

Proteins were induced for purification from pET vectors (Novagen – EMD Millipore) in BL21(DE3) [91] backgrounds as 2 L cultures that were grown to OD_600_∼0.7 at 30°C, at which point 1 mM IPTG was added. (Volumes in this and subsequent steps were scaled down by a factor of 10 for copurification assays). Cultures were left overnight at 30°C to induce protein and were harvested in the morning by spinning in a Beckman J2-21 centrifuge with a JA-17 rotor at 7,000 rpm. Cell pellets were then washed in buffer (50 mM sodium phosphate pH 8.0; 300 mM NaCl), re-centrifuged, and stored as cell pellets at −80°C.

For purifications, cell pellets were thawed on ice, resuspended in 30 mL of the sodium phosphate wash buffer (except for His-tagged Kil, see below) with 1 mg/mL lysozyme and an EDTA-Free c0mplete Protease Inhibitor Cocktail Tablet (Roche), and incubated on ice for 30 minutes. Cells were disrupted by sonication on ice (Branson Sonifier 250; 50% level, output 4) in a series of six alternating 30-second periods of sonication and 30-second rest periods. Cell lysates were clarified of debris by spinning at 40,000 rpm at 4°C for 45 minutes in an 80 Ti rotor with an Optima XL-100K Ultracentrifuge (Beckman).

*E. coli* FtsZ was purified from WM971 cell lysates by successive 20% and 30% ammonium sulfate cuts. Following the second cut, protein was resuspended in polymerization buffer (50 mM MES pH 6.5; 50 mM KCl; 2.5 mM MgCl_2_; 1 mM EGTA; 10% sucrose), flash-frozen in liquid nitrogen and stored at −80°C.

His-tagged ZipA_C_ proteins were purified by gravity flow over water-washed and buffer-equilibrated cobalt-conjugated resin with a 5 mL bed volume at 4°C. Imidazole at pH 8.0 was added to 10 mM in cell lysates for loading onto the column. Following loading, columns were washed successively in ten-column volumes of purification buffer (50 mM HEPES pH 7.5; 300 mM NaCl) with 10 mM, 25 mM, and 50 mM imidazole. His-tagged ZipA_C_s were eluted in 10 mL purification buffer+250 mM imidazole. Eluted proteins were concentrated and subjected to buffer exchange into polymerization buffer using Amicon Ultra-10K Centrifugal Filter Devices (Millipore), flash-frozen in liquid nitrogen, and stored at −80°C.

His_6_-FLAG-Kil was primarily induced in, and purified from, BL21(DE3) *ftsA_R286W_ ΔzipA::aph* pBS58 cells. The exception was for copurification experiments, where the fusion was also prepared from *zipA_WT_^+^* or *zipA_L286Q_* ^+^ backgrounds, as noted. Following induction, His_6_-FLAG-Kil was found to be predominantly associated in insoluble inclusion bodies, leading to very low yields under native conditions, even in an *ftsA_R286W_ ΔzipA::aph* background. We therefore purified His_6_-FLAG-Kil under denaturing conditions according to the QIAexpressionist Handbook (QIAGEN, 2003) protocol, followed by renaturation by dialysis. Purity was estimated at >95% by Coomassie staining.

Lysates from His_6_-FLAG-Kil-expressing cells were prepared similarly as described above, except cell pellets were resuspended in denaturing lysis buffer (100 mM sodium phosphate, pH 8.0, 10 mM Tris-Cl, 6M guanidine hydrochloride) with an EDTA-Free c0mplete Protease Inhibitor Cocktail Tablet (Roche), but without lysozyme. After incubation for 30 minutes at room temperature, cells were sonicated as described above.

These His_6_-FLAG-Kil-containing lysates were then loaded by gravity flow onto water-washed and denaturation lysis buffer-equilibrated cobalt-conjugated resin with a 5 mL bed volume at room temperature. Columns were washed in ten-column volumes of freshly prepared denaturing wash buffer (100 mM sodium phosphate, pH 6.3, 10 mM Tris-Cl, 8 M urea). Denatured His_6_-FLAG-Kil was eluted in 10 mL freshly prepared denaturing elution buffer (100 mM sodium phosphate, pH 4.5, 10 mM Tris-Cl, 8 M urea). The eluted protein was then renatured by dialysis into polymerization buffer overnight and through the following day (three 2 L changes of buffer in total). Renatured His_6_-FLAG-Kil was concentrated using Amicon Ultra-3K Centrifugal Filter Devices (Millipore), flash-frozen in liquid nitrogen, and stored at −80°C.

### Interaction and sedimentation assays

To assay interaction between purified proteins, 200 mL samples were prepared in polymerization buffer containing 6 µM bovine serum albumin. FtsZ at 5 µM, His_6_-FLAG-Kil at 10 µM, and/or His_6_-tagged ZipA_C_ domains (WT and L286Q) at 5 µM were included as indicated. 50 µL of 50% buffer-equilibrated α-FLAG M2 affinity resin (Sigma-Aldrich) were added and samples were incubated at room temperature, mixing, for one to three hours for binding. Samples were then loaded onto gravity flow columns and the resin was washed with 25 mL polymerization buffer. Following washes, resin was recovered in 250 µL buffer, SDS-PAGE sample buffer was added and samples were boiled and separated by 20% SDS-PAGE followed by Coomassie blue staining.

Sedimentation assays were performed essentially as previously described [66] in a Beckman TL-100 Ultracentrifuge, but using a TLA 100.3 rotor at 70,000 rpm with appropriate adaptors and speed-resistant tubes (Beckman). 100 µL samples were prepared in polymerization buffer with FtsZ at 5 µM, His_6_-FLAG-Kil at 10 µM, His_6_-tagged ZipA_C_ domains (WT and L286Q) at 5 µM, and GTP at 1 mM. Components were added in the following order: polymerization buffer, Kil buffer or Kil, FtsZ, ZipA_C_, and GTP. For reactions with calcium-induced bundling, CaCl_2_ was added to 1 mM after GTP addition.

### GTPase activity assay

GTPase activities were determined using the EnzChek Phosphate Assay Kit (Molecular Probes) in reactions set up with the same concentrations and buffers as for sedimentation assays, but in a 96 well plate and with the required purine nucleoside phosphorylase enzyme and 7-Methyl-6-thio-D-guanosine (MESG) substrate components. Reactions were initiated by adding one half the reaction volume as buffer with FtsZ alone, simultaneously via multi-channel pipette, into the other half of the reaction volume that contained all other components. (For reactions without FtsZ, buffer with GTP alone was used to simultaneously initate reactions). OD_360_ readings were taken every 30 seconds using a Synergy Mx Microplate Reader (BioTek). GTP hydrolysis rates were calculated based on a phosphate standard curve.

### Phage one-step growth assay

One-step growth was done according to Frank Stahl (unpublished). Briefly, 10 mL MG1655 was growth at 39°C in tryptone broth to OD_600_ = 0.4 (∼1.5×10^8^ per mL). The cells were pelleted and suspended in TMG (see above) and incubated at 37°C for 30 minutes to starve the cells. NaCN was added at 2×10^−3^ M and 1.8 mL was dispensed to two small glass-plating tubes. To initiate the growth curve, 0.2 mL phage stock at 1.5×10^8^ per mL was added to the cell suspension. Phage adsorption was monitored over a 30 minute period by mixing 0.1 mL infected cells into 4.9 mL tryptone broth containing 0.25 mL chloroform and plating appropriate dilutions on C600 host cells. After a 30 minute adsorption the infected cells were diluted 100-fold into tryptone broth and two further dilutions were made into 39°C tryptone broth, one 100-fold and one 5×10^3^-fold. At various times, 0.1 mL samples were taken from these dilutions and plated immediately on C600.

## Supporting Information

Table S1 Oligonucleotides used in this study.
(DOCX)
