# Supplementary material for: The Kil Peptide of Bacteriophage λ Blocks Escherichia coli Cytokinesis via ZipA-Dependent Inhibition of FtsZ Assembly
Source: PLoS Genet. 2014 Mar 20;10(3):e1004217. doi: 10.1371/journal.pgen.1004217 (PMC3961180; doi:10.1371/journal.pgen.1004217)
Supplement: Table S1 — Oligonucleotides used in this study. (DOCX) [file pgen.1004217.s001.docx]

Table S1. Oligonucleotides used in this study

| Name | | Sequence (5’ to 3’) | | Use | |
| --- | --- | --- | --- | --- | --- |
| AW24 | | AAT TAC GTG ACG GAT GGA AAC | | 21 base forward oligo to sequence *kil* deletion | |
| DPH170 | | GTT GAA GCT TTT AGT GAA TGC TTT TGC TTG ATC TCA G | | 37 base reverse *kil* oligo with *Hind*III site | |
| DPH199 | | GTA ACT GCA GAT GGA TCA AAC ACT TAT GGC TAT CCA G | | 37 base forward *kil* oligo with *Pst*I site | |
| DPH200 | | GAT CAC TAG TTT AGT GAA TGC TTT TGC TTG ATC TCA G | | 37 base reverse *kil* oligo with *Spe*I site | |
| DPH202 | | GAT CCT GCA GTT AGT GAA TGC TTT TGC TTG ATC TCA G | | 37 base reverse *kil* oligo with *Pst*I site | |
| DPH217 | | GAT CCC CGG GAG GAG GAA TTC ACC ATG GAT CAA ACA CTT ATG GCT ATC CCA | | 51 base forward *kil* oligo (with ribosome binding site) with *Xma*I site | |
| DPH309 | | GAC GAT GAC AAA ATC CAG CGT ATG GAT CAA ACA CTT ATG GCT ATC CAG AC | | 50 base forward *kil* oligo to add half of 5’ *his_6_-flag* coding sequence | |
| DPH310 | | GAT AGG ATC CGA CTA CAA GGA CGA CGA TGA CAA AAT CCA GCG TAT G | | 46 base forward *kil* oligo to add remaining half of 5’ *his_6_-flag* coding sequence with *BamH*I site | |
| JS308 | | GAA AAG ATG TTT CGT GAA GCC GTC GAC GCT TAG AAA AAA TGG ATA TTA ATA CTG AAA CTG AGA TCA AGC A | | 70 base oligo to make *kil_tyr31UAG_* | |
| LT199 | | CCT GAC GTC TAA GAA ACC ATT ATT ATC | | 27 base forward oligo to sequence pJ10 insert | |
| LT200 | | AAC GAC AGG AGC ACG ATC ATG CG | | 24 base reverse oligo to sequence pJ10 insert | |
| LT793 | | TCC AGA CTA AAT TCA CTA TCG CCA CTT TTA TTG GCG CCG TCG ACG CTT ATA AAA AAT GGA TAT TAA TAC T | | 70 base hybrid oligo containing seven codon deletion in *kil*, paired with LT795 to make a PCR product containing *kil* deletion | |
| LT795 | | GAA TGC TTT TGC TTG ATC TCA G | | 22 base reverse oligo in *gam*, paired with LT793, also used to sequence *kil* deletion | |
| MH23 | | CTC AGA CAT TAC GGT GCG TAC GTC TGC AAA GTC C**G**C GTT CT CAA ACC CGG ACG AGT AAT CAG TTC AGC G | | 70 base oligo to make *ftsZ*_v208A_ (bold) in the *mutS* mutant strain, AW65. | |
| MH24 | | ATC CAG CAG GGA GAT ACC GCG GCC CAG AAC TTT C**C**G CAG TTT GTC GTT CGG GAT AGT GAT CAG AGA GTC C | | 70 base oligo to make *ftsZ*_L169R_ (bold) in a *mutS* mutant strain, AW65. | |
| MH36 | | GCC CAC TTC ATC GGC AAT ATG **T**TG **T**GC **G**GA **C**TG C**T**G CAT CAG CTT GAA GTT CTG CAG CTC GTC ACC GTA A | | 70 base oligo to make *zipA*_L286Q_ (underlined and bold) with 4 additional wobble changes (bold) for high frequency recombination in MMR^+^ cells. | |
| MH38 | | AAT ATG **T**TG **T**GC **G**GA **C**TG C**T** | | 20 base oligo to identify *zipA*_L286Q_ along with oligo MH41 | |
| MH39 | | GAA TTT CCG GCG CAT CCA TC | | 20 base oligo to make PCR product with MH60. *zipA* product for sequencing. | |
| MH41 | | GTC GTT CGC AGT TCT GTC AG | | 20 base oligo 3' of *zipA* | |
| MH52 | | ACA AGC GGG GGT TCG AAG AG | | 20 base forward oligo to amplify *zipA* for mutagenic PCR. | |
| MH53 | | TGT TTG CCG TGG TGG CAA GG | | 20 base reverse oligo to amplify *zipA* for mutagenic PCR. | |
| MH58 | | CAC GAC ACC GCC CAC TTC ATC GGC AAT ATG **T**TG **T**GC **G**GA **C**TG C**C**G CAT CAG CTT GAA GTT CTG CAG CTC G | | 70 base oligo to make *zipA*_L286R_ (underlined and bold) with 4 additional wobble changes (bold) for high frequency recombination in MMR^+^ cells. | |
| MH59 | | C AAT ATG **T**TG **T**GC **G**GA **C**TG C**C** | | 21 base oligo to identify *zipA*_L286R_ along with oligo MH41 | |
| MH60 | | CGC AAT GGA CAG TTA GGA TAT G | | 22 base oligo to make PCR product with MH39. *zipA* product for sequencing. | |
| MH61 | | ACA GCA AAA AGC ACG ATT TCA TC | | 23 base oligo for sequencing *zipA* PCR product. | |
| MH62 | | TGC TGA ATG CAC AGG CTG CG | | 20 base oligo for sequencing *zipA* PCR product. | |
| MH63 | | TGC AGC AGC CTG CCT ATC AG | | 20 base oligo for sequencing *zipA* PCR product. | |
| MH82 | | AAG CAC GAC ACC GCC CAC TTC ATC GGC AAT ATG C**C**G **T**GC **C**GA **C**TG **A**AG CAT CAG CTT GAA GTT CTG CAG | | 69 base oligo to make *zipA_Q290R_* with 4 additional wobble changes (bold) for high frequency recombination in MMR^+^ cells. | |
| MH83 | | TGA TGC T**T**C A**G**T C**G**G C**A**C **G** | | 19 base oligo to identify *zipA*_Q290R_ along with oligo MH41 | |
| NC820 | | CAG CAT CAG TTG GCT CGT GG | | 20 base forward oligo to make PCR product with NC821 to sequence *ftsZ* | |
| NC821 | | CCC GTC GCC TGA ACG ATA CG | | 20 base reverse oligo to make PCR product with NC820 to sequence *ftsZ* | |
| WM268 | | GTC TCG AGG GCG TTG GCG TCT TTG AC | | 26 base reverse oligo to amplify *zipA* with *Xho*I site | |
| WM269 | | AAG GAT CCG ATA AAC CGA AGC GCA AAG AAG CGG | | 33 base forward oligo to amplify 3’ (C-terminal) portion of *zipA* with *BamH*I site | |
| WM356 | | CCA AGC TTT ATC CTC CGA ACA AGC GTT TG | | 29 base reverse oligo to amplify *minD* with *Hind*III site | |
| WM960 | | GCC GTC GAC GGA GGT GGC GGA GGC GCA CGC ATT ATT GTT GTT AC | | 44 base forward oligo to amplify *minD* with *Sal*I site | |
| XMZ325 | | AGG CAC AAA TCG GAG AGA AAC T | | 22 base forward oligo to amplify *ftsZ* for mutagenic PCR. | |
| XMZ326 | | GAA ACC CAA ATT CCA GTC AAT TC | | 23 base reverse oligo to amplify *ftsZ* for mutagenic PCR. | |
| XMZ340 | | ACT CTC TGA TCA CTA TCC CG | | 20 base internal forward oligo to sequence *ftsZ* | |
| XMZ341 | | ACG CCA GAA CCC ATC ATT GC | | 20 base internal reverse oligo to sequence *ftsZ* | |
| XT96 | | TGA TGC AAT TTC TAT GCG CAC C | | 22 base forward oligo to sequence pNB15 insert | |
| XT97 | | ATA CCC ACG CCG AAA CAA GC | | 20 base reverse oligo to sequence pNB15 insert | |
